# Isolation and Structural Characterization of Two New Xanthone Derivatives from the Marine-Derived Fungus *Penicillium* sp. F28

**DOI:** 10.3390/molecules31213820

**Published:** 2026-10-23

**Authors:** Ailiman Abulaizi, Jingzhe Yuan, Zijun Xiong, Shiqing Zhang, Rong Wang, Shuai Ma, Zhikai Guo

**Affiliations:** 1 Morphological Center, School of Basic Medical Sciences, Xinjiang Medical University, Urumqi 830017, China; 2 Hainan Key Laboratory of Tropical Microbe Resources, Institute of Tropical Bioscience and Biotechnology, Chinese Academy of Tropical Agricultural Sciences Key Laboratory for Biology and Genetic Resources of Tropical Crops of Hainan Province, Hainan Institute for Tropical Agricultural Resources, Haikou 571101, China; yuanjingzhe@catasitbb.cn (J.Y.); xiongzijun@catasitbb.cn (Z.X.); zhangshiqing@catasitbb.cn (S.Z.); 3 Hainan Provincial Key Laboratory of Tropical Maricultural Technologies, Hainan Academy of Ocean and Fisheries Sciences, Haikou 571126, China; wangrong1982@gmail.com

**Keywords:** marine fungi, *Penicillium* sp. F28, xanthones, isolation and structural elucidation

## Abstract

Eight compounds, including two new compounds, xishaxanthone A (**1**) and xishaxanthone B (**2**), and six known compounds (**3**–**8**), were isolated from the marine-derived fungus *Penicillium* sp. F28 and structurally characterized. Xishaxanthone A (**1**) is a new xanthone derivative which possesses a 1,7-dihydroxanthone skeleton with a methyl carboxylate group at C-8, a substitution pattern rarely encountered among xanthone natural products. The structure was determined by a combination of techniques including HR-ESI-MS (High-Resolution Electrospray Ionization Mass Spectrometry), NMR (Nuclear Magnetic Resonance), and ECD (Electronic Circular Dichroism) calculations. Furthermore, a hypothetical biosynthetic pathway for compound **1** is proposed.

## 1. Introduction

Nature generates an immense array of chemical compounds and bioactive molecules whose structural sophistication far surpasses human design capabilities. Many of these natural products have been developed into commercially available pharmaceuticals or have served as promising starting points in drug discovery [1]. It is estimated that natural products and their derivatives account for a substantial proportion of approved drugs, particularly in the fields of anti-infective and anticancer therapies, underscoring their enduring value as a source of therapeutic leads. Covering more than 70% of the Earth’s surface, the ocean is among the largest and most under-explored ecosystems. In contrast to land-based habitats, marine systems feature extreme physicochemical parameters like elevated salinity, fluctuating pressure, low temperature, and scarce nutrients [2]. These harsh and highly variable conditions impose strong selective pressure on marine organisms, driving the evolution of specialized metabolic pathways that are often absent in terrestrial counterparts. To adapt to extreme environments and counteract potential threats in the deep sea, marine organisms biosynthesize a diverse array of active secondary metabolites which possess novel and complex structures [3,4,5]. As a result, the chemical space explored by marine organisms remains largely distinct from that of terrestrial natural products, offering unique opportunities for the discovery of compounds with unprecedented scaffolds and modes of action.

The remarkable structural novelty of marine natural products is exemplified by xanthones, which are among the most structurally versatile and biologically active secondary metabolites produced by marine-derived fungi. Xanthones, a class of oxygen-containing heterocycles built on a dibenzo-*γ*-pyrone scaffold, exhibit a highly adaptable structural framework that allows diverse chemical modifications, strongly influencing their physicochemical and bioactive profiles [6]. The xanthone core can be decorated through prenylation, hydroxylation, halogenation, and glycosylation, generating a wide range of derivatives with markedly different lipophilicity, membrane permeability, and target affinity. This structural plasticity not only underlies the diverse bioactivities of xanthones but also provides a rational basis for structure–activity relationship studies and lead optimization. Extensive research in recent years has revealed a broad spectrum of biological activities associated with xanthones, including anti-HIV [7,8], anticancer [9], enzyme inhibitory [10,11] activities, making them attractive candidates for drug discovery and development [12,13]. Nevertheless, the chemical space of marine-derived xanthones has been only partially explored, with most studies focusing on a limited set of fungal strains and known compounds. Continued efforts in isolating and characterizing novel xanthone derivatives are therefore essential for expanding the structural diversity of this family, and for providing new chemical entities for future functional studies. Structural analysis of natural products remains challenging due to their inherent complexity and limited availability. Particularly, the structural elucidation of trace natural products is often hindered by the limitations of conventional methods. Inspired by recent advances in complementary structural techniques such as Microcrystal Electron Diffraction (MicroED) [14], which enables the direct determination of structures from minute amounts of powder, there is a pressing need to explore novel bioactive architectures from untapped natural sources.

As part of our ongoing investigation into bioactive metabolites from marine organisms of the South China Sea [15,16,17], which has previously yielded a series of structurally diverse and biologically active secondary metabolites, the metabolically prolific fungal strain *Penicillium* sp. F28 was isolated from seawater collected from the Xisha Islands. This region is characterized by unique hydrodynamic conditions and a highly diverse marine microbial community, providing a promising reservoir for the discovery of novel natural products. Subsequent chemical exploration of its fermentation extract led to the identification of two new compounds (**1** and **2**), together with six known natural products (**3**–**8**). The new compounds (**1** and **2**) represent new additions to the xanthone family, further expanding the chemical diversity of marine-derived fungal metabolites. Herein, we report the isolation and structural elucidation of these compounds, along with an evaluation of their biological activity.

## 2. Results

**Figure 1:**
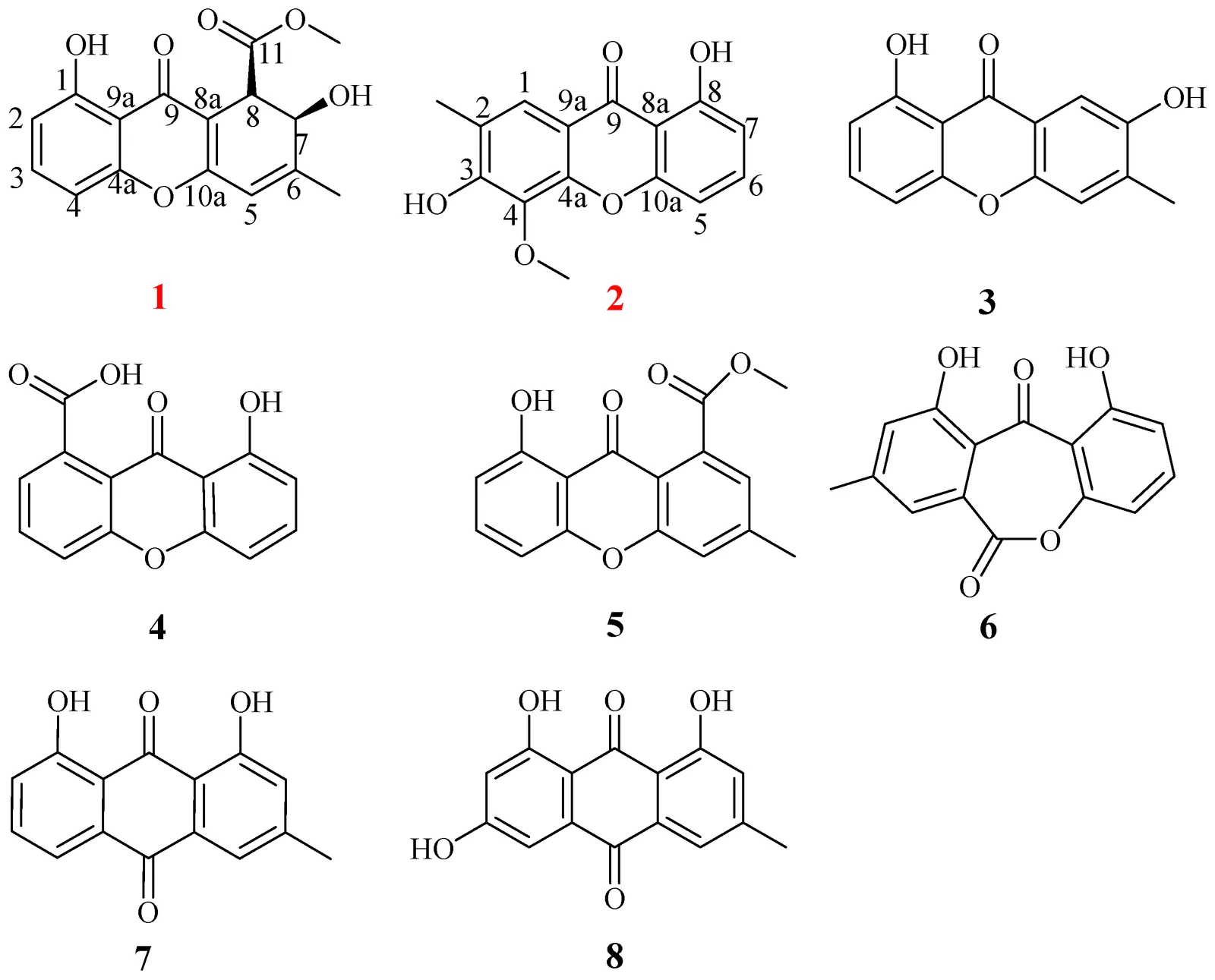
Chemical structures of compounds **1**–**8**. The numbers of new compounds are shown in red.

EtOAc (Ethyl Acetate) extract of the fungus *Penicillium* sp. F28 was subjected to repeated CC (Column Chromatography) and semi-preparative HPLC (High-Performance Liquid Chromatography) to yield two new compounds, xishaxanthone A (**1**), xishaxanthone B (**2**), along with six known compounds, 1,7-dihydroxy-6-methylxanthone (**3**) [18], monodictyxanthone (**4**) [19], 8-hydroxy-3-methyl-9-oxo-9*H*-xanthene-1-carboxylic acid methyl ester (**5**) [20], monodictylactone (**6**) [21], chrysophanol (**7**) [22] and emodin (**8**) [23]. These known compounds were identified by comparison of their experimental spectroscopic data with those in the literature (Figure 1).

### 2.1. Structure Elucidation

Xishaxanthone A (**1**) was obtained as yellow needles. Its molecular formula was determined as C_16_H_14_O_6_ based on a positive HR-ESI-MS peak at *m*/*z* 303.0865 [M + H]^+^ (calcd. for C_16_H_15_O_6_, 303.0854), consistent with ten degrees of unsaturation (Figure S7). The UV (Ultraviolet Spectroscopy) spectrum showed four absorption maxima (237, 269, 299, and 347 nm), implying the presence of a xanthone chromophore. The ^1^H NMR spectrum revealed the existence of one methyl proton at *δ*_H_ 2.14 (3H, t, *J* = 1.4 Hz, 6-CH_3_), one methoxy proton at *δ*_H_ 3.71 (3H, s, 11-OCH_3_), one phenolic hydroxyl as a doublet at *δ*_H_ 4.07 (1H, d, *J* = 10.1 Hz, 7-OH), four olefinic protons at *δ*_H_ 6.06 (1H, m, H-5), 6.79 (1H, dd, *J* = 8.3, 0.8 Hz, H-2), 6.86 (1H, dd, *J* = 8.4, 0.8 Hz, H-4), 7.49 (1H, t, *J* = 8.3 Hz, H-3), and one phenolic hydroxyl proton at *δ*_H_ 12.58 (1H, s, 1-OH) (Figure S1, Table 1). The DEPTQ135 NMR spectrum indicated the existence of one methyl group at *δ*_C_ 19.8 (6-CH_3_), one methoxy group at *δ*_C_ 52.7 (11-OCH_3_), six methines at *δ*_C_ 40.4 (C-8), 70.5 (C-7), 107.0 (C-4), 111.5 (C-2), 116.2 (C-5) and 135.0 (C-3), and eight quaternary carbons at *δ*_C_ 110.1 (C-8a), 110.5 (C-9a), 155.5 (C-4a), 157.9 (C-6), 160.5 (C-1), 160.6 (C-10a), 172.7 (C-11), 180.2 (C-9) (Figure S2, Table 1).

**Figure 2:**
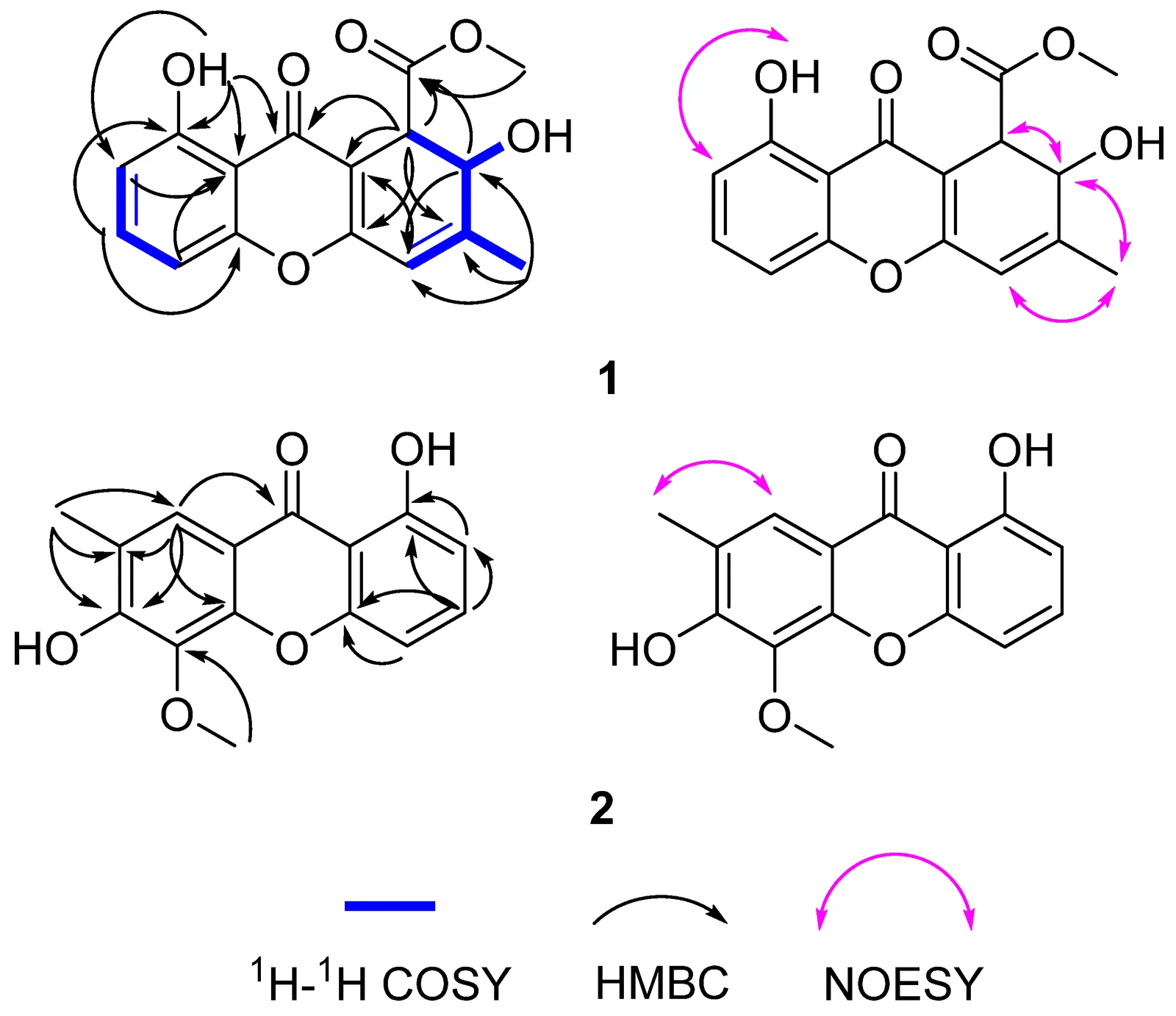
Key HMBC, ^1^H-^1^H COSY and ROESY correlations of new compounds **1** and **2**.

The following structural fragments were established through ^1^H-^1^H COSY (^1^H-^1^H Correlation Spectroscopy) data (Figure S5) which enabled the establishment of two discrete spin systems [H-2–H-3–H-4 and H-5–CH_3_-6–H-7–H-8], as shown in Figure 2. The positions of the methyl and methoxy groups in compound **1** were revealed by the key HMBC (Heteronuclear Multiple-Bond Correlation) correlations from 6-CH_3_ to C-5, C-6, and C-7; and from 11-OCH_3_ to C-11. The planar structure was also proposed by the HMBC correlations from 1-OH to C-1, C-2, C-9, and C-9a, from H-3 to C-1 and C-4a, from H-5 to C-8a, from H-7 to C-5 and C-11, and from H-8 to C-6, C-8a, C-9, C-10a and C-11 (Figure S4).

**Figure 3:**
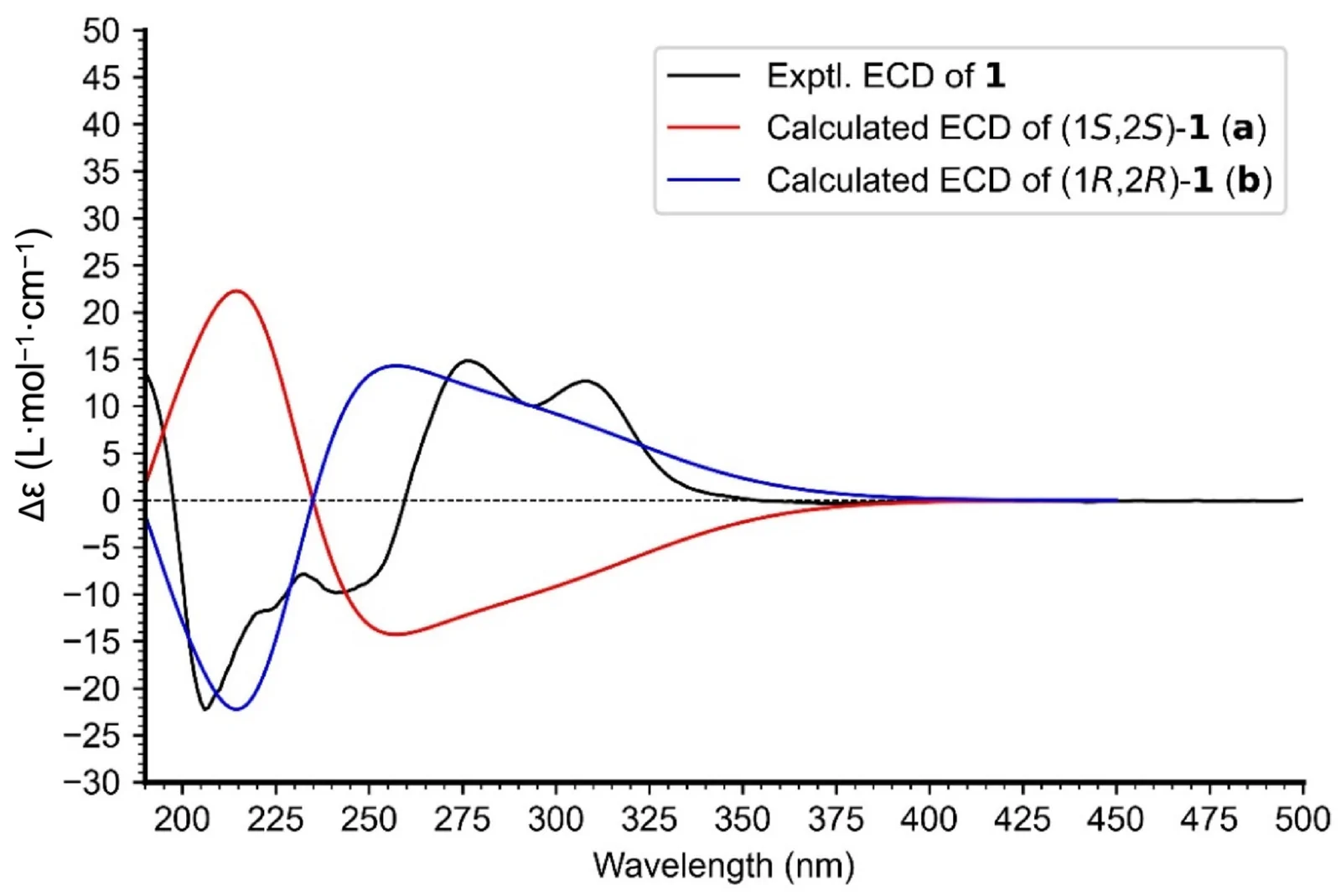
Experimental and calculated ECD spectra of new compound **1**.

The relative configuration of compound **1** was determined via NOESY (Nuclear Overhauser Effect Spectroscopy) analysis. Cross-peaks between OH-1/H-2, between H-7/H-8, and between 6-CH_3_/H-5, were observed (Figure S6), indicating the above structural deduction. The absolute configurations of C-7 and C-8 were both determined as *R* configurations by ECD calculations (Figure 3 and Supporting Information, Part II).

Xishaxanthone B (**2**) was obtained as colorless amorphous solid. Its molecular formula was determined to be C_15_H_12_O_5_ based on the HR-ESI-MS ion at *m*/*z* 271.0627 [M − H]^−^ (calcd. for C_15_H_11_O_5_, 271.0611), indicating ten degrees of unsaturation (Figure S15). The ^1^H NMR spectrum revealed the existence of one singlet methyl proton at *δ*_H_ 2.35 (3H, s, 2-CH_3_), one singlet oxymethyl proton at *δ*_H_ 3.83 (3H, s, 4-OCH_3_), and four olefinic protons at *δ*_H_ 7.68 (1H, t, *J* = 8.3 Hz, H-6), 7.50 (1H, s, H-1), 7.03 (1H, d, *J* = 8.3 Hz, H-5), 6.77 (1H, d, *J* = 8.0 Hz, H-7), which suggested a tetrasubstituted xanthone skeleton (Figure S10). The DEPTQ135 NMR (Distortionless Enhancement by Polarization Transfer with Quaternary detection, 135°pulse) spectrum contained 15 resonances resulting from one carbonyl (*δ*_C_ 180.4, C-9), eight sp^2^-hybridized methine carbons (*δ*_C_ 167.8, C-4; 160.8, C-8; 151.9, C-3; 139.5, C-2; 136.8, C-6; 119.5, C-1; 109.6, C-7; 106.9, C-5), four quaternary carbon atoms (*δ*_C_ 155.4, C-10a; 148.0, C-4a; 115.3, C-9a; 107.8, C-8a), one oxygenated sp^3^-hybridized methyl (*δ*_C_, 52.0, 4-OCH_3_), and one methyl carbon (*δ*_C_,17.7, 2-CH_3_) (Table 1). DEPTQ135 NMR shift values of *δ*_C_ 51.9 corresponded to oxygenated sp3-hybridized methyl groups, the HMBC correlations from 4-OCH_3_ to C-4 revealed its position. The planar structure was established by the HMBC correlations from H-1 to C-3 and C-9, from 2-CH_3_ to C-1–3, and from H-6 to C-8 and C-10a (Figure 2). In addition, NOESY correlations of 2-CH_3_ with H-1 confirmed the assignment of the structure for **2**. Finally, the whole interpretation of 1D and 2D NMR data allowed confirmation of the complete structure of **2** shown in Figure 1. Compound **2** is a new tetrasubstituted xanthone.

### 2.2. The Proposed Biosynthetic Pathway of New Compound ***1***

**Scheme 1:**
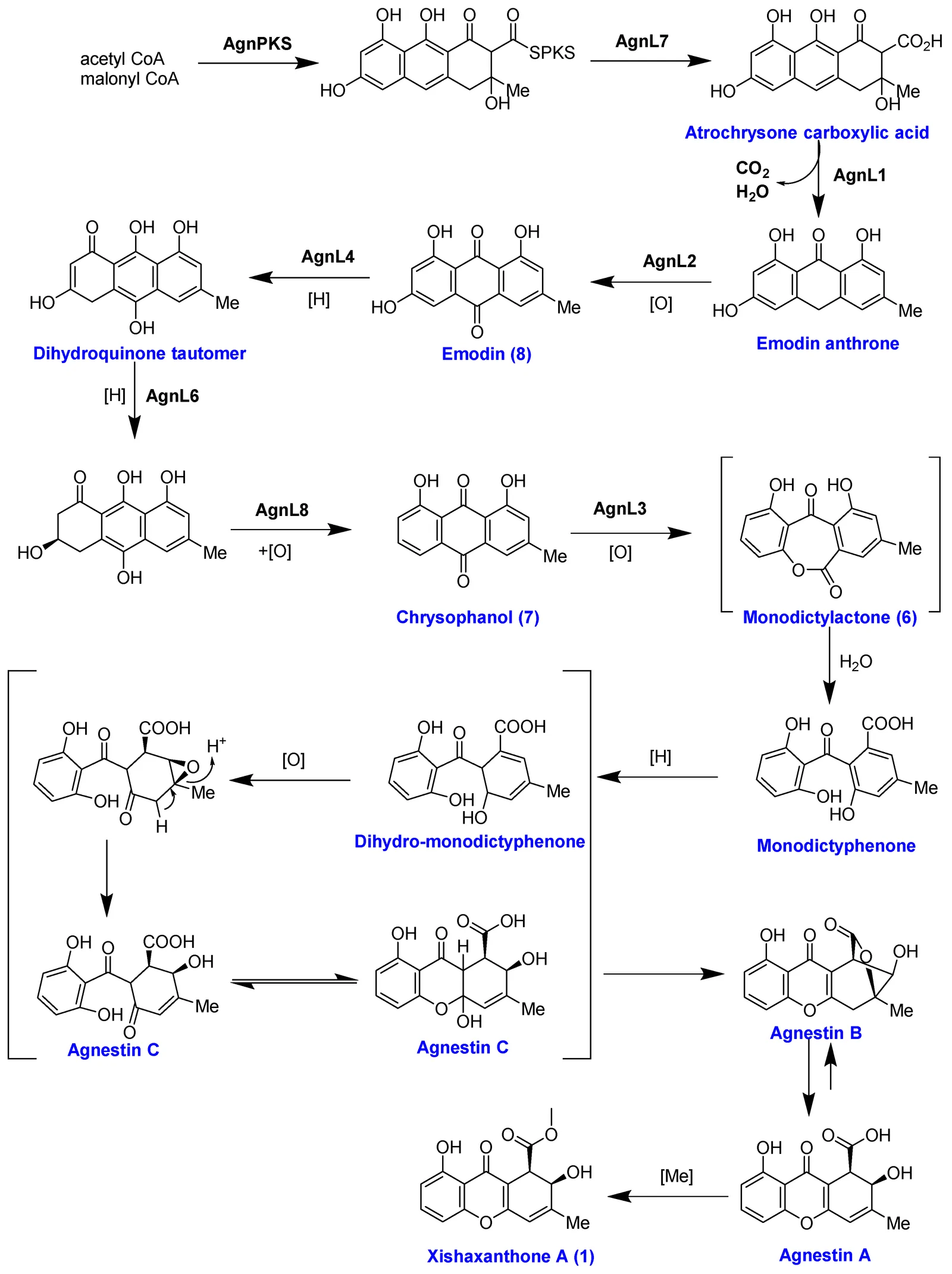
Proposed biosynthetic pathway for new compound **1**.

Drawing on earlier reports in the literature [24], a unified biosynthetic route to xishaxanthone A (**1**) is presented in Scheme 1. The pathway is initiated by AgnPKS (=MdpG), which catalyzes the assembly and cyclization of an octaketide tethered to the PKS as a thioester. This intermediate is subsequently hydrolyzed by AgnL7 (=MdpF) to release atrochrysone carboxylic acid as the first enzyme-free intermediate. AgnL1 then mediates a concerted decarboxylation elimination step, converting this acid into emodin anthrone, which is oxidized to emodin by AgnL2 (=MdpH1). Reduction of emodin, catalyzed by AgnL4 (=MdpK), affords the corresponding dihydroquinone tautomer. This tautomer acts as the substrate for AgnL6 (=MdpC), whose reductive action yields a further reduced product; subsequent dehydration by AgnL8 (=MdpB), followed by spontaneous autoxidation, leads to chrysophanol. Its conversion into agnestins A and B proceeds via reduction to dihydromonodictyphenone, oxidation to agnestin C, and direct rearrangement to either agnestin A or B. Additional transformations could ultimately produce xishaxanthone A. It should be emphasized that these steps are speculative and are proposed chiefly to rationalize the observed structural relationships among the isolated natural products (Scheme 1), representing a hypothesis based on established biosynthetic logic that requires further in vivo/vitro validation.

## 3. Discussion

In the ongoing search for structurally diverse and biologically active secondary metabolites in the South China Sea, two new compounds were isolated from the culture of *Penicillium* sp. F28. Their structures, including their absolute configurations, were unequivocally established by comprehensive spectroscopic analyses (HR-ESI-MS, 1D/2D NMR) in conjunction with ECD calculations. Notably, compound **1** is a new xanthone derivative featuring a 1,7-dihydroxanthone skeleton with a methyl carboxylate moiety at C-8, constituting the first example of this substitution pattern in xanthone natural products [25,26].

In light of previous studies [24], a plausible biosynthetic pathway for compound **1** is proposed in Scheme 1. It is hypothesized that compound **1** is biosynthetically derived from a PKS-assembled octaketide chain via a sequence of cyclization, oxidative modifications, and rearrangement, a speculation strongly corroborated by the co-isolation of biogenetic congeners including emodin, chrysophanol, and agnestins A–C (Scheme 1). This pathway not only rationalizes the structural features of compound **1** but also provides a valuable clue for future genome-mining of the related biosynthetic gene clusters in this strain.

**Figure 4:**
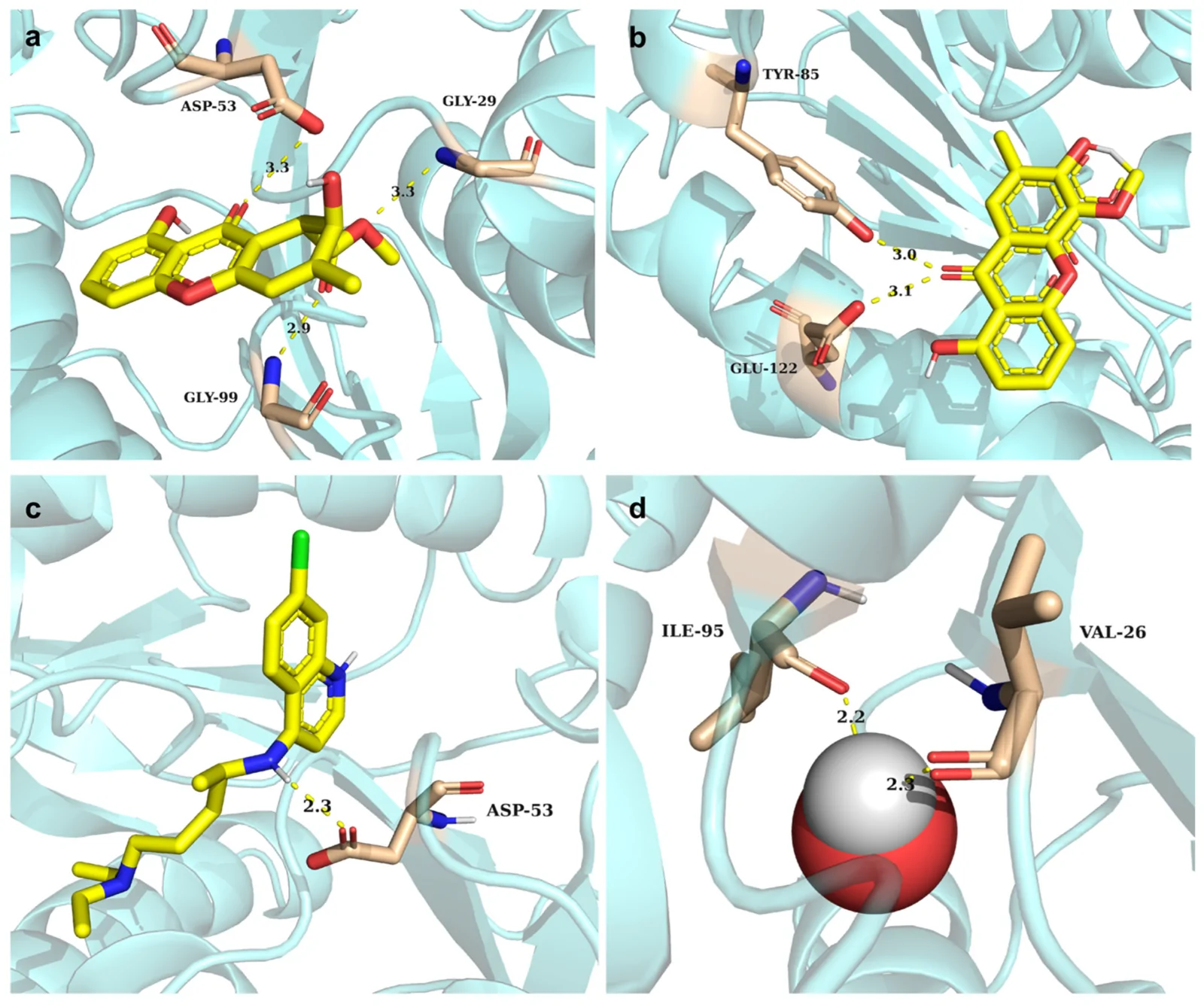
Predicted binding modes and H-bond interactions of two new compounds with *Pf*LDH enzyme. The backbone of the *Pf*LDH enzyme is presented in a cyan ribbon model, and all hydrogen bonding residues are shown as stick models and labeled by heteroatoms. The compounds are labeled by heteroatoms as follows: yellow for C, grey for H, red for O, and green for Cl. Yellow dashed lines represent hydrogen bond interactions and represent bond length in angstroms (Å). (**a**) Xishaxanthone A (**1**). (**b**) Xishaxanthone B (**2**). (**c**) Chloroquine (positive control). (**d**) Water (negative control).

In the present study, tested compounds showed no antimicrobial activity against the selected pathogenic strains. In contrast to the synthetic chlorinated xanthones reported by Resende et al., our new compounds lack antibacterial activity, consistent with recent SAR studies on xanthone derivatives [27]. It has been established that potent antimicrobial activity in xanthones requires specific structural determinants, such as optimized halogen/hydroxy/methoxy substitutions that enable multifaceted mechanisms of action including cell wall disruption and DNA synthesis inhibition [28]. Our compounds possess a 1,7-dihydroxyxanthone skeleton bearing a C-8 methyl carboxylate moiety and thus lack the key pharmacophores described above. In addition, the known compound 1-hydroxy-6-methyl-8-(hydroxymethyl)xanthone, which has a hydroxyl group at the 7-position and a CH_2_OH group at the 8-position and is structurally similar to the new compound **1**, was found to be active against *Eurotium repens* (inhibition zone 2 mm) and *Ustilago violacea* (inhibition zone 2 mm) according to [29]. Although the corresponding activity of the new compound **1** was not screened in the present study, it is plausible that the substituents at these two positions influence its activity. Compound **2**, given the promising antimalarial activity reported for structurally related molecules [30], was further subjected to molecular docking analysis against the target protein to preliminarily evaluate its potential antimalarial properties (Figure 4). It differs from the previously reported compound 1-hydroxy-5,6,7-trimethoxyxanthone, which exhibited the highest effect with good activity (IC_50_ = 9.57 µM), by only two substituents (a hydroxyl group at the 3-position and a methoxy group at the 2-position in the former versus two methoxy groups in the latter). Compound **1** was docked in parallel for comparison.

In this docking conformation (Figure 4a), the yellow small molecule ligand binds to the pocket region of the protein, forming a total of three hydrogen-bond interactions with the Asp-53, Gly-29, and Gly-99 residues, with hydrogen-bond lengths of 3.3 Å, 3.3 Å, and 2.9 Å, respectively; among them, the 2.9 Å one is a relatively strong hydrogen bond. The ligand is anchored within the protein binding cavity through the hydrogen-bond network. The amino acid residue Asp-53 provides a polar side chain to participate in hydrogen bond formation, while the two glycines Gly-29 and Gly-99 establish hydrogen bonds with the ligand through backbone atoms. The docking binding free energy is −7.5 kcal/mol, suggesting that this binding mode has a favorable thermodynamic tendency; glycine has no side chain and little steric hindrance, which facilitates the stable placement of the ligand within the pocket, and the polar side chain of Asp-53 further stabilizes the ligand-protein complex. This optimal docking conformation (Figure 4b) has a binding energy of −8.1 kcal/mol, which is superior to the binding mode in Figure 4a. The ligand forms hydrogen bonds with two amino acid residues, Tyr-85 and Glu-122, with hydrogen bond distances of 3.0 Å and 3.1 Å, respectively. The phenolic hydroxyl group of Tyr-85 and the carboxyl side chain of Glu-122 jointly participate in the formation of hydrogen bonds, constraining the small molecule within the protein pocket. Tyr-85 can also provide an aromatic ring to participate in potential hydrophobic/π-related interactions, while the charged side chain of Glu-122 strengthens polar interactions; the lower binding energy indicates that the protein–ligand complex is more stable under this conformation, making it optimal docking pose. These favorable interactions suggest that compound **2** is highly likely to possess antimalarial activity. To validate the docking protocol, chloroquine (positive control) exhibited an optimal binding energy of −7.1 kcal/mol with *Pf*LDH, demonstrating strong binding affinity. Conversely, the negative control (water) showed a significantly weaker binding energy of −1.7 kcal/mol, confirming the discriminatory power of the scoring function.

## 4. Materials and Methods

### 4.1. Fungal Materials

The fungal strain of *Penicillium* sp. F28 was isolated from seawater collected from the Xisha Islands in the South China Sea and identified as *Penicillium* sp. by internal transcribed spacer (ITS) sequence analysis (GenBank accession number: QB196990). The voucher specimen of fungal strain *Penicillium* sp. F28 was deposited in the Hainan Key Laboratory of Tropical Microbe Resources, Institute of Tropical Bioscience and Biotechnology, Haikou, China.

### 4.2. General Experimental Procedures

NMR spectra were recorded on a Bruker AVANCE III 500 MHz spectrometer (Bruker, Karlsruhe, Germany). HRESIMS data were determined on an Agilent 6210 TOF LC-MS mass spectrometer (Agilent, Santa Clara, CA, USA). Optical rotation value was recorded by a JASCO P-1020 digital polarimeter (JASCO, Tokyo, Japan). UV and IR data were measured on a UV-2550 spectrometer (Shimadzu, Kyoto, Japan) and a Nicolet 380 infrared spectrometer (Thermo Electron Corporation, Madison, WI, USA), respectively. The ECD data were collected on a JASCO J-715 spectropolarimeter (JASCO, Tokyo, Japan). The semi-preparative HPLC was conducted on a Waters 1525 HPLC equipped with a XBridge C_18_ column (5 μm, 250.0 mm × 10.0 mm; Waters Corporation, Milford, MA, USA). Thin-layer chromatography (TLC) was performed on pre-coated glass plates (silica gel GF254, Qingdao Marine Chemical Inc., Qing-dao, China). Column chromatography (CC) was performed on silica gel (45–75 μm; Qing-dao Marine Chemical Inc., Qingdao, China), ODS (40–60 μm; Osaka Soda Co., Ltd., Hyogo, Japan) and Sephadex LH-20 (Cytiva, Uppsala, Sweden).

### 4.3. Fermentation, Extraction, and Isolation

The strain was cultivated on potato dextrose agar (PDA, Guangdong Huankai Microbial SCI. and Tech. CO., LTD, Guangdong, China) at 28 °C for 4 d. Then, the agar plugs with mycelia were added to ME medium (malt extract 10.0 g/L, sucrose 10.0 g/L, tryptone 1.0 g/L) cultured on a rotary shaker (160 rpm) at 28 °C for 3 d to afford a seed culture. The seed culture was then inoculated into rice solid medium (a total of 200 1-L Erlenmeyer flasks were prepared, each containing 90.0 g rice and 0.1 L seawater) and fermented at 28 °C for 30 d under static conditions.

The fungal culture was exhaustively extracted using EtOAc to obtain a crude extract (90.0 g); this was then fractionated via silica gel vacuum liquid chromatography with the elution gradient of chloroform (CHCl_3_)/methanol (MeOH) (100:1, 50:1, 25:1, 10:1, 5:1, 2:1, 1:1, 0:1) to yield five fractions [Fractions (Frs.) 1–5].

Fr. 2 was purified via CC over RP-C18 eluting with a MeOH−H_2_O gradient (from 5:5 to 1:0) to obtain four subfractions (Fr. 2-1 to 2-4). Fr. 2-2 was purified by Sephadex LH-20 (MeOH) and then further purified using semi-preparative HPLC (60% MeOH−H_2_O) to obtain **1** (3.1 mg, t*_R_* 55.0 min), (45% MeOH−H_2_O) to obtain **2** (2.2 mg, t*_R_* 35 min).

### 4.4. Identification of New Compounds

Xishaxanthone A (**1**)

Yellow needles. ${\left[\alpha \right]}_{D}^{25}$ +88.0, (*c* 0.5, CH_3_OH); UV (CH_3_OH) *λ*_max_ (log ε) 223 (2.07), 237 (1.95), 269 (2.46), 299 (1.91), 347 (2.05) nm (Figure S8); ECD (CH_3_OH) *λ*_max_ (Δε) 242 (−5.88), 277 (+8.84), 308 (+7.57) nm; IR (Infrared Spectroscopy) (kBr) *ν*_max_ 3403, 1728, 1648, 1472, 1233, 1052 cm^−1^ (Figure S9); HRESIMS *m*/*z*: 303.0865 [M + H]^+^, calcd for C_16_H_15_O_6_, 303.0854 (Figure S7); 1D and 2D NMR data, see Table 1 and Supporting Information (Figures S1–S6).

Xishaxanthone B (**2**)

Colorless amorphous solid. UV (MeCN) *λ*_max_ (log ε) 268 (2.72), 279 (2.51), 330 (1.69), 380 (2.43) nm (Figure S16); IR (kBr) *ν*_max_ 3486, 3356, 2932, 1647, 1540, 1470, 1236 cm^−1^ (Figure S17); HRESIMS *m*/*z*: 271.0627 [M + H]^−^, calcd for C_15_H_11_O_5_, 271.0611 (Figure S15); 1D and 2D NMR data, see Table 1 and Supporting Information (Figures S10–S14).

### 4.5. Computational Methods of ECD

A Conformer-Rotamer Ensemble Sampling Tool (CREST) [31,32] was utilized to generate candidate conformers, and DFT calculations were performed using the Gaussian 16 program [33]. Conformers within an energy window of 10 kcal/mol were optimized at a B3LYP/6-31G(d) level of theory with Grimme’s D3 dispersion correction (“Empirical Dispersion = GD3” key words in input files). Frequency analysis of all optimized conformations was undertaken at the same level of theory to ensure they were true local minima on the potential energy surface. Then, energies of all optimized conformations were evaluated by M062X/6-311 + G (2d, p) with D3 dispersion correction. The Gibbs free energy of each conformer was calculated by adding “Thermal correction to Gibbs Free Energy” obtained by frequency analysis to electronic energies obtained at M062X/6-311 + G (2d, p). Room-temperature (298.15 K) equilibrium populations were calculated according to the Boltzmann distribution law. Those conformers accounting for over 1% of population were subjected to subsequent calculations.

NMR shielding constants were calculated with the GIAO (Gauge-Including Atomic Orbital) method at mPW1PW91-SCRF/6-31 + G (d, p) level with IEFPCM solvent model in chloroform solvent. The shielding constants obtained were converted into chemical shifts by referencing to TMS (Tetramethylsilane) at 0 ppm (*δ*_cal_ = *σ*_TMS_ − *σ*_cal_), where the *σ*_TMS_ was the shielding constant of TMS calculated at the same level [34]. The DP4^+^ probabilities of each possible candidate were calculated with the Excel spreadsheet provided by Sarotti et al. [35]. For each possible candidate, the parameters *a* and *b* of the linear regression *δ*_cal_ = *aδ*_exp_ + *b*; the correlation coefficient, *R*^2^; the mean absolute error (MAE) defined as Σn |*δ*_cal_ − *δ*_exp_|/n; and the corrected mean absolute error, CMAE, defined as Σn |*δ*_corr_ − *δ*_exp_|/n, where *δ*_corr_ = (*δ*_cal_ − b)/a, were calculated.

TDDFT (Time-Dependent Density Functional Theory) ECD calculations were run at CAM-B3LYP/6-311G(d) level of theory in MeOH with the IEFPCM solvent model. For each conformer, 36 excited states were calculated [36]. The calculated ECD curves were generated using Multiwfn 3.6 software [37].

### 4.6. Evaluation of Bioactivity

The antimicrobial activities of these metabolites were evaluated using previously reported methods [38]. New compounds were evaluated against ten bacteria (*E. coli*, methicillin-resistant *Staphylococcus aureus*, *Micrococcus luteus*, *Xanthomonas oryzae pv*. *oryzae*, *Photobacterium damselae*, *Vibrio alginolyticus*, *Vibrio owensii*, *Vibrio harveyi*, *Streptococcus agalactiae*, *Ralstonia solanacearum*). The bacteria were cultured in LB (Luria-Bertani) medium (10.0 g of peptone,15.0 g of sodium chloride, 3.0 g of yeast extract, and 1.0 L of distilled H_2_O) at 28·°C (160 rpm) for 24 h. Each compound was tested at 10 mg/mL in DMSO (Dimethyl Sulfoxide) solvent. A DMSO solution was used as a negative control, and kanamycin solution (10 mg/mL) was used as a positive control. Each test was performed in triplicate, with incubation at 28 °C for 12 h. The size of the inhibition zone was then recorded. However, none of the tested compounds showed any activity.

### 4.7. Molecular Docking

The crystal structure of *Plasmodium falciparum* lactate dehydrogenase (*Pf*LDH) was downloaded from the Protein Data Bank (PDB ID: 1LDG). The receptor structure was prepared using AutoDockTools 1.5.7: water molecules were removed, polar hydrogens were added, and Kollman charges were assigned. Two new compounds isolated from *Penicillium* sp. F28 were used as ligands for the docking study. The initial 3D structures of the compounds were generated using HyperChem Professional 8.0 (Hypercube Inc., Gainesville, FL, USA) and geometrically optimized using the semiempirical PM3 method. Subsequently, Gasteiger charges and non-polar hydrogens were assigned using AutoDockTools to generate the appropriate PDBQT files for docking.

A grid box was defined to encompass the active site of 1LDG, with center coordinates of X = 25.729 Å, Y = 16.305 Å, Z = 38.292 Å and dimensions of X = 62.65 Å, Y = 51.71 Å, Z = 55.69 Å. Molecular docking was performed using AutoDock Vina with default parameters. The binding energy (affinity, kcal/mol) was calculated for the top-ranked conformation. The 3D binding modes were visualized using PyMOL 3.1.8.

## 5. Conclusions

In summary, this study reports the isolation and structural characterization of two new compounds (**1**, **2**), and six known compounds (**3**–**8**) from the marine-derived fungus *Penicillium* sp. F28, obtained from the Xisha Islands. A hypothetical biosynthetic pathway for the new compound **1** is proposed.

The discovery of these new xanthones not only expands the chemical diversity of fungal-derived natural products, but also provides novel scaffolds for the development of drug leads. Although conventional NMR and HR-ESI-MS were sufficient for structural elucidation in this study, the structural characterization of trace natural products often poses significant challenges, particularly when they are obtained as amorphous powders and fail to yield single crystals suitable for X-ray diffraction. In such cases, emerging complementary techniques such as Microcrystal Electron Diffraction (MicroED) offer a powerful alternative [14]. MicroED enables direct determination of the absolute configurations and crystal structures of natural products from minute amounts of powder without the need for extensive purification or crystallization [39]. Integrating such cutting-edge techniques in future studies will greatly enhance the speed and accuracy of characterizing novel xanthone derivatives. In conclusion, this study enriches the chemical diversity of marine fungal metabolites, and provides a solid foundation for the future development of novel xanthone-based drugs.

## Figures and Tables

**Table 1:** ^1^H (500 MHz), and ^13^C (125 MHz) data of compounds **1** and **2** (*δ* in ppm, *J* in Hz).

Position	1 ^a^		2 ^b^	
	*δ*_C_	*δ*_H_ (*J* in Hz)	*δ*_C_	*δ*_H_ (*J* in Hz)
1	160.5		119.5	7.50 (s)
2	111.5	6.79 (dd, 8.3, 0.8)	139.5	
3	135.0	7.49 (t, 8.3)	151.9	
4	107.0	6.86 (dd, 8.4, 0.8)	167.8	
4a	155.5		148.0	
5	116.2	6.06 (m)	106.9	7.03 (d, 8.3)
6	157.9		136.8	7.68 (t, 8.3)
7	70.5	4.74 (t, 8.4)	109.6	6.77 (d, 8.0)
8	40.4	4.24 (d, 7.1)	160.8	
8a	110.1		107.8	
9	180.2		180.4	
9a	110.5		115.3	
10a	160.6		155.4	
11	172.7			
1-OH		12.58 (s)		
2-CH_3_			17.7	2.35 (s)
4-OCH_3_			52.0	3.83 (s)
6-CH_3_	19.8	2.14 (t, 1.4)		
7-OH		4.07 (d, 10.1)		
11-OCH_3_	52.7	3.71 (s)		

^a^ Acquired in CDCl_3_; ^b^ Acquired in DMSO-*d*_6_.

## Data Availability

The data that support the findings of this study are available from the corresponding authors upon reasonable request.

## Supplementary Materials

The following supporting information can be downloaded at https://www.mdpi.com/article/10.3390/molecules31213820/s1.

## Abbreviations

The following abbreviations are used in this manuscript:

NMR	Nuclear Magnetic Resonance
CC	Column Chromatography
HPLC	High-Performance Liquid Chromatography
EtOAc	Ethyl Acetate
HR-ESI-MS	High-Resolution Electrospray Ionization Mass Spectrometry
DEPTQ135	Distortionless Enhancement by Polarization Transfer with Quaternary detection, 135° pulse
HMBC	Heteronuclear Multiple Bond Correlation
^1^H-^1^H COSY	^1^H-^1^H Correlation Spectroscopy
NOESY	Nuclear Overhauser Effect Spectroscopy
ECD	Electronic Circular Dichroism
LB	Luria Bertani
DMSO	Dimethyl Sulfoxide
TMS	Tetramethylsilane
TDDFT	Time Dependent Density Functional Theory
GIAO	Gauge-Including Atomic Orbital
IR	Infrared Spectroscopy
UV	Ultraviolet Spectroscopy
