# Supplementary material for: Isolation and Structural Characterization of Two New Xanthone Derivatives from the Marine-Derived Fungus Penicillium sp. F28
Source: Molecules. 2026 Oct 23;31(21):3820. doi: 10.3390/molecules31213820 (PMC13764920; doi:10.3390/molecules31213820)
Supplement: Supplementary file 1 [file molecules-31-03820-s001.zip › molecules-4558496-supplementary.pdf]

Isolation and Structural Characterization of Two New Xanthone Derivatives from the Marine-Derived Fungus *Penicillium* sp. F28

Ailiman Abulaizi [1,*], Jingzhe Yuan [2], Zijun Xiong [2], Shiqing Zhang [2], Rong Wang [3], Shuai Ma [2,*] and Zhikai Guo [2,*]

[1] Morphological Center, School of Basic Medical Sciences, Xinjiang Medical University, Urumqi 830017, China; helime5052@163.com (A.A.)

[2] Hainan Key Laboratory of Tropical Microbe Resources, Institute of Tropical Bioscience and Biotechnology, Chinese Academy of Tropical Agricultural Sciences, Key Laboratory for Biology and Genetic Resources of Tropical Crops of Hainan Province, Hainan Institute for Tropical Agricultural Resources, Haikou 571101, China; yuanjingzhe@catasitbb.cn (J.Y.); xiongzijun@catasitbb.cn (Z.X.); zhangshiqing@catasitbb.cn (S.Z.)

[3] Hainan Provincial Key Laboratory of Tropical Maricultural Technologies, Hainan Academy of Ocean and Fisheries Sciences, Haikou 571126, China; wangrong1982@gmail.com (R.W.)

* Correspondence: helime5052@163.com (A.A.); mashuai@catasitbb.cn (S.M.); guozhikai@catasitbb.cn (Z.G.)

Supporting Information

Part I

Figure S1. ^{1}H NMR (500 MHz, chloroform-*d*) spectrum of compound **1**
Figure S2. DEPTQ135 (125 MHz, chloroform-*d*) spectrum of compound **1**
Figure S3. HSQC spectrum of compound **1**
Figure S4. HMBC spectrum of compound **1**
Figure S5. ^{1}H-^{1}H COSY spectrum of compound **1**
Figure S6. NOESY spectrum of compound **1**
Figure S7. The HRESIMS spectrum of the new compound **1**
Figure S8. UV spectrum of compound **1**
Figure S9. IR spectrum of compound **1**
Figure S10. ^{1}H NMR (500 MHz, DMSO-d_6) spectrum of compound **2**
Figure S11. DEPTQ135 (500 MHz, DMSO-d_6) spectrum of compound **2**
Figure S12. HSQC spectrum of compound **2**
Figure S13. HMBC spectrum of compound **2**
Figure S14. NOESY spectrum of compound **2**
Figure S15. The HRESIMS spectrum of the new compound **2**
Figure S16. UV spectrum of compound **2**
Figure S17. IR spectrum of compound **2**

Figure S1. ^{1}H NMR (500 MHz, chloroform-*d*) spectrum of compound **1**

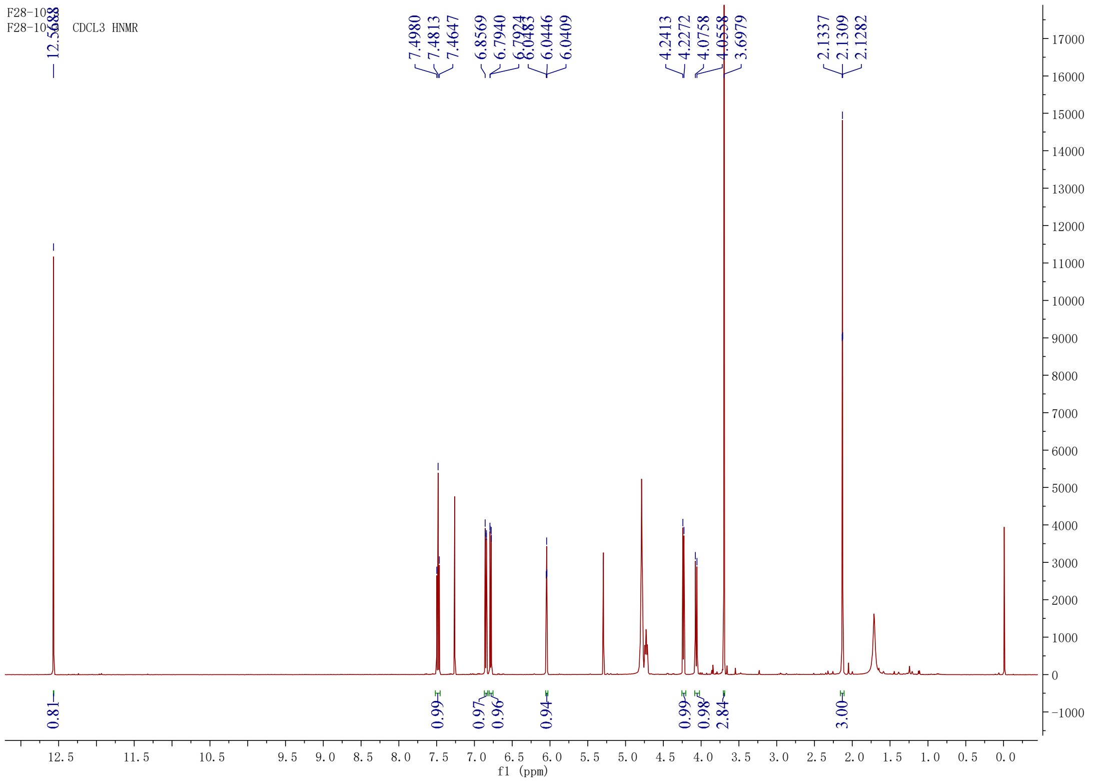

Figure S2. DEPTQ135 (125 MHz, chloroform-*d*) spectrum of compound **1**

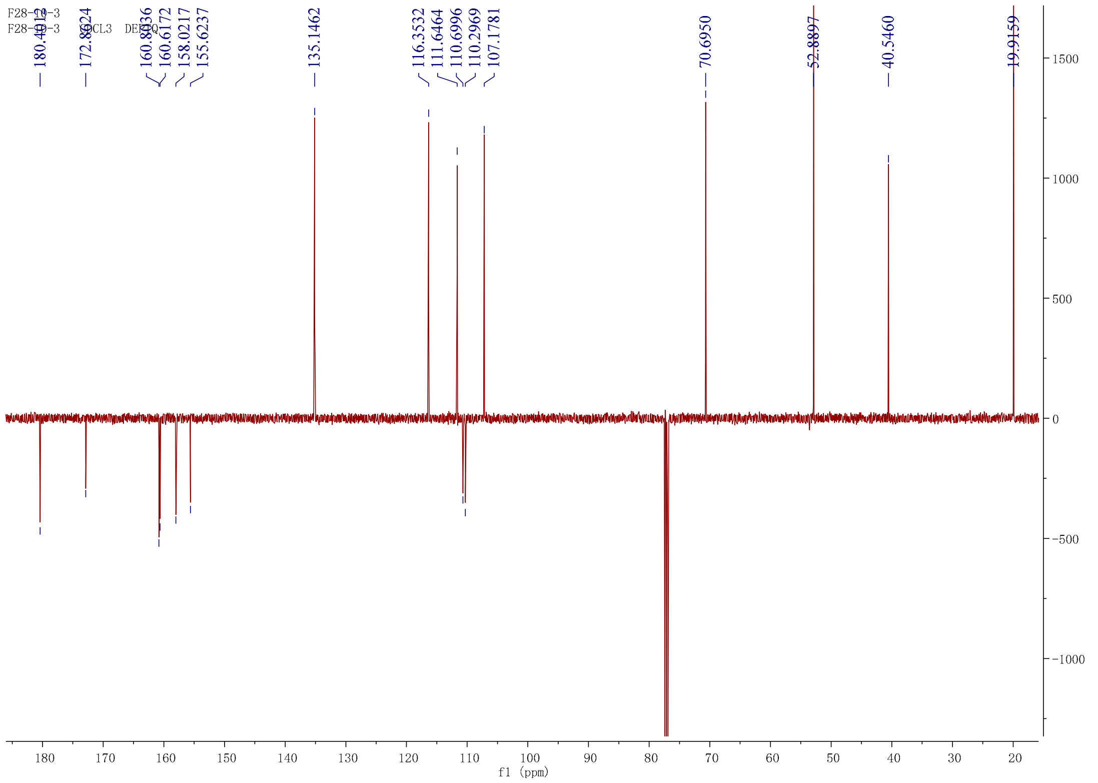

Figure S3. HSQC spectrum of compound **1**

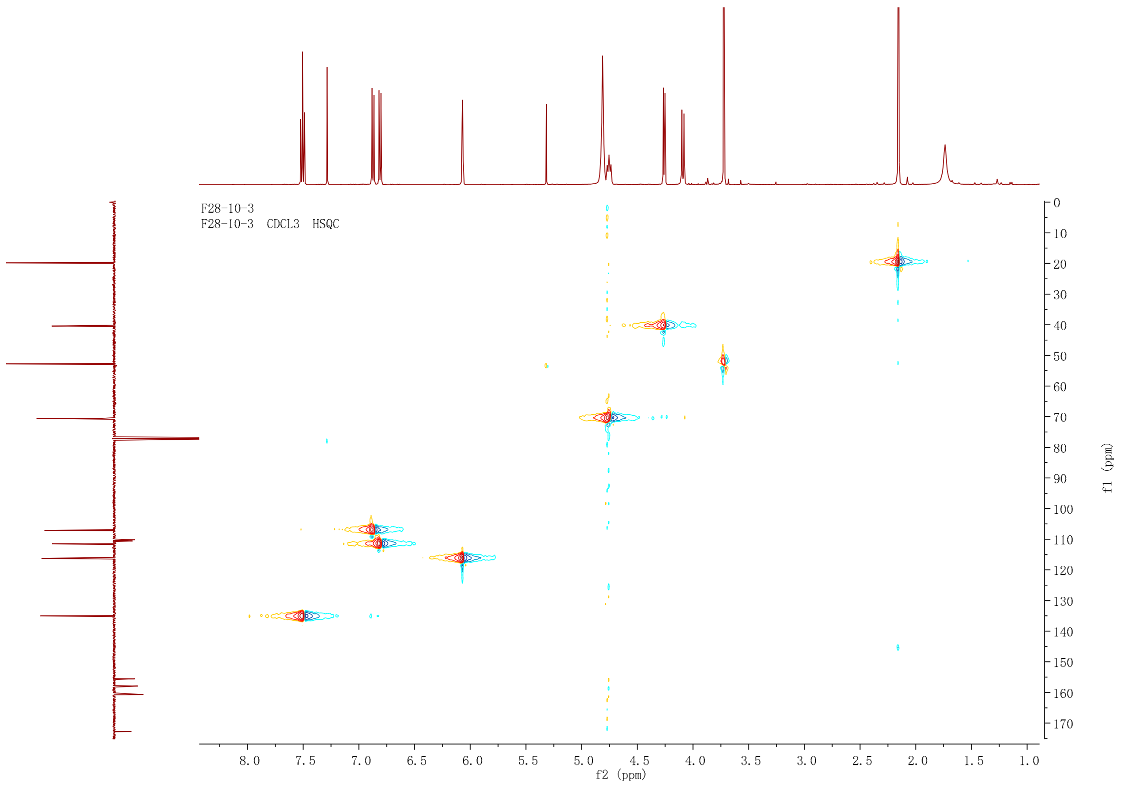

Figure S4. HMBC spectrum of compound **1**

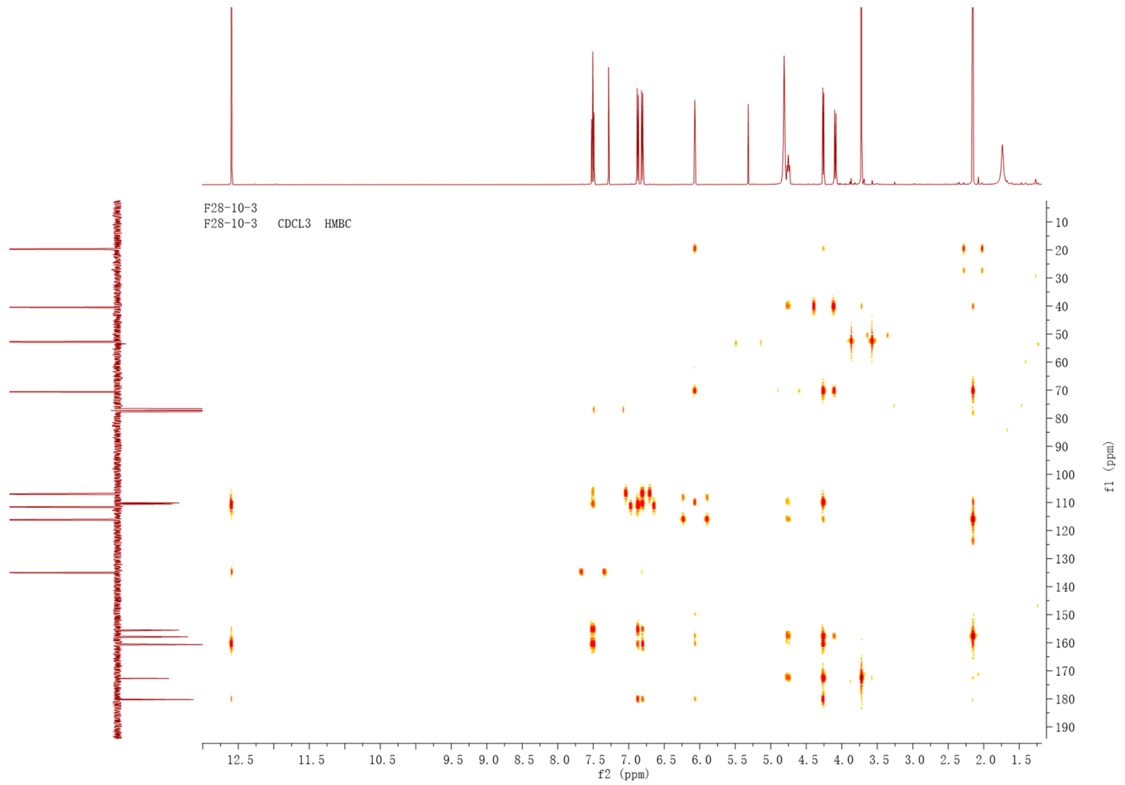

Figure S5. ^{1}H-^{1}H COSY spectrum of compound **1**

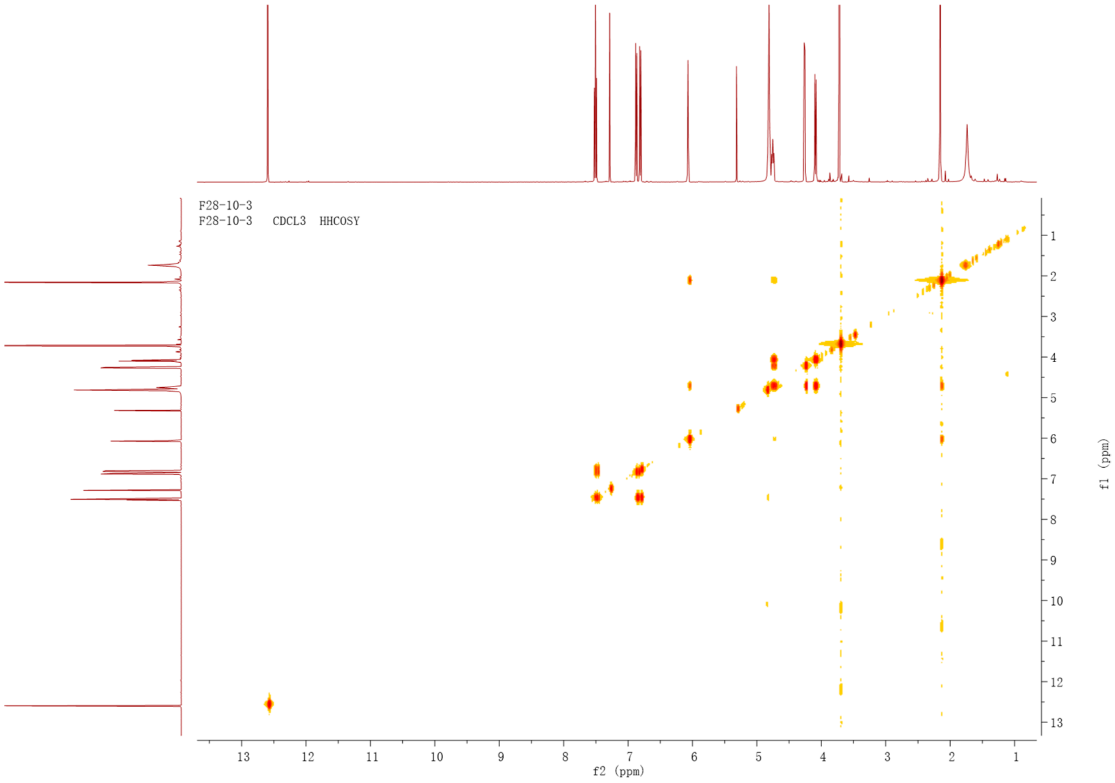

Figure S6. NOESY spectrum of compound **1**

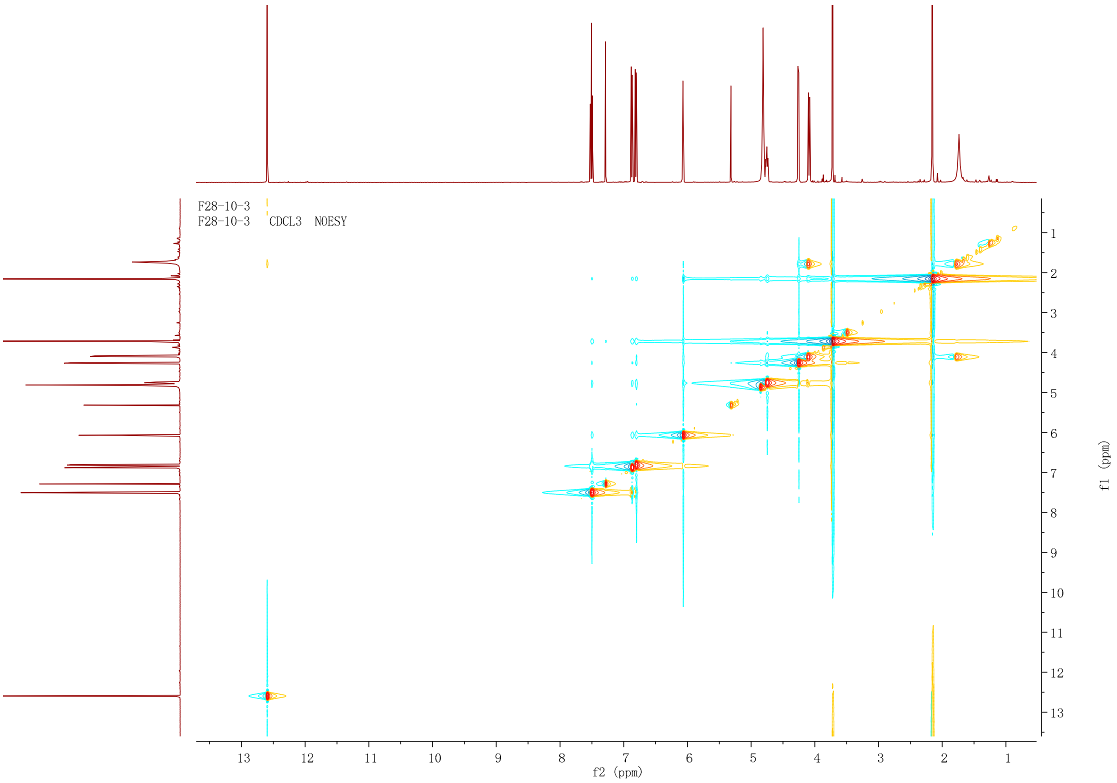

Figure S7. The HRESIMS spectrum of the new compound **1**

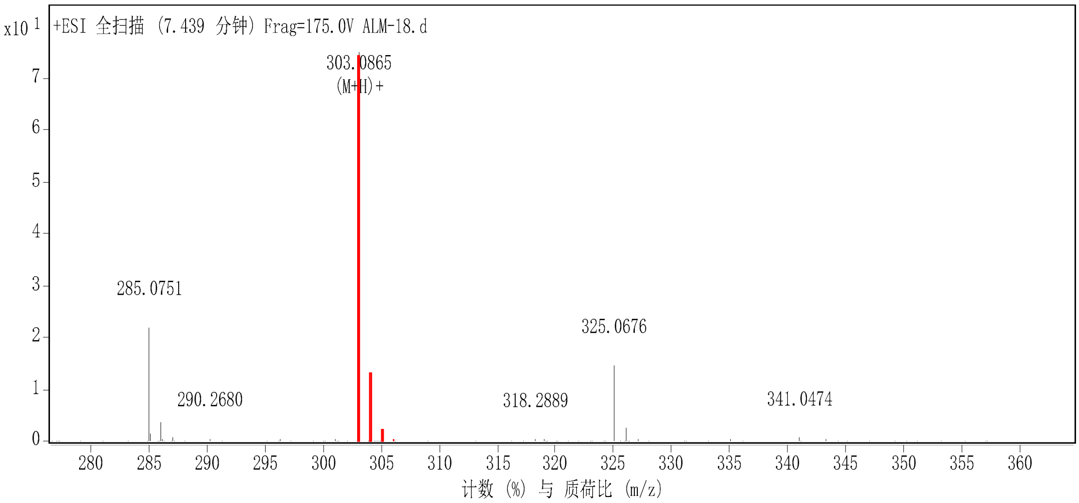

Figure S8. UV spectrum of compound **1**

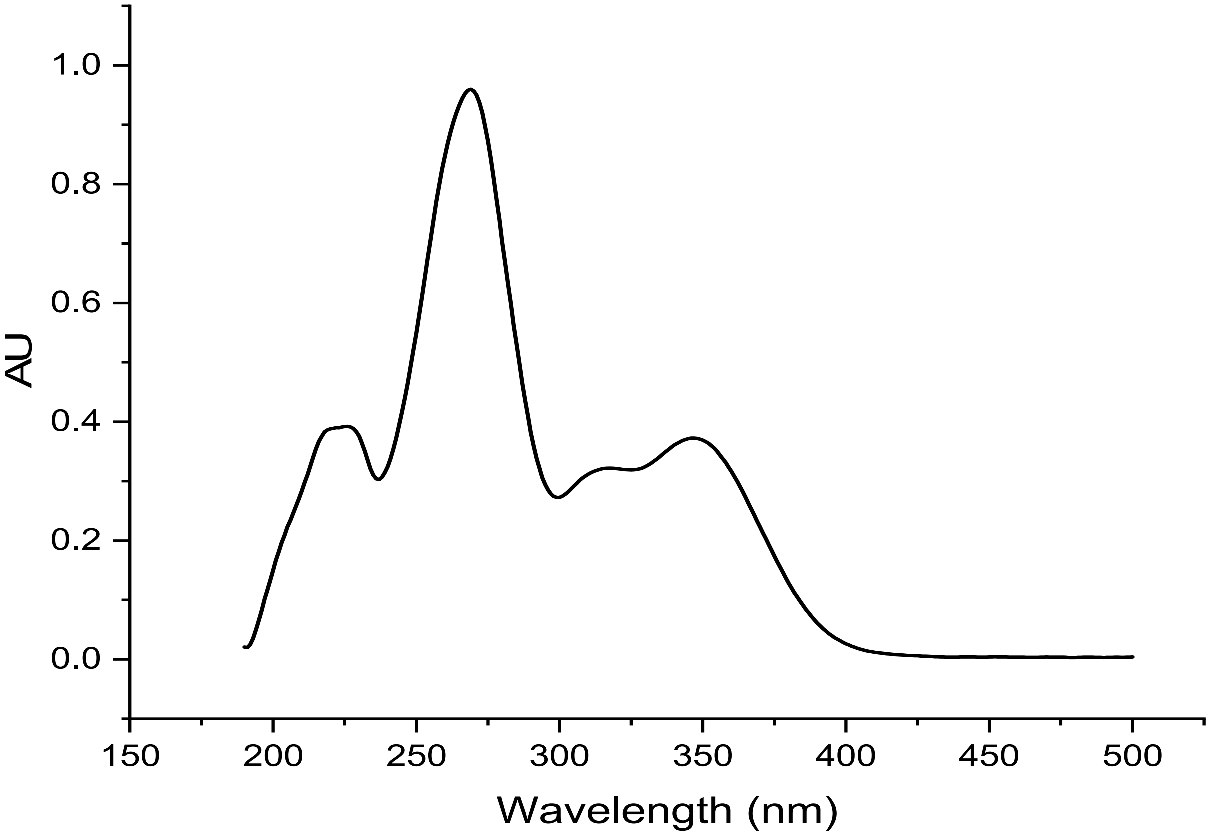

Figure S9. IR spectrum of compound **1**

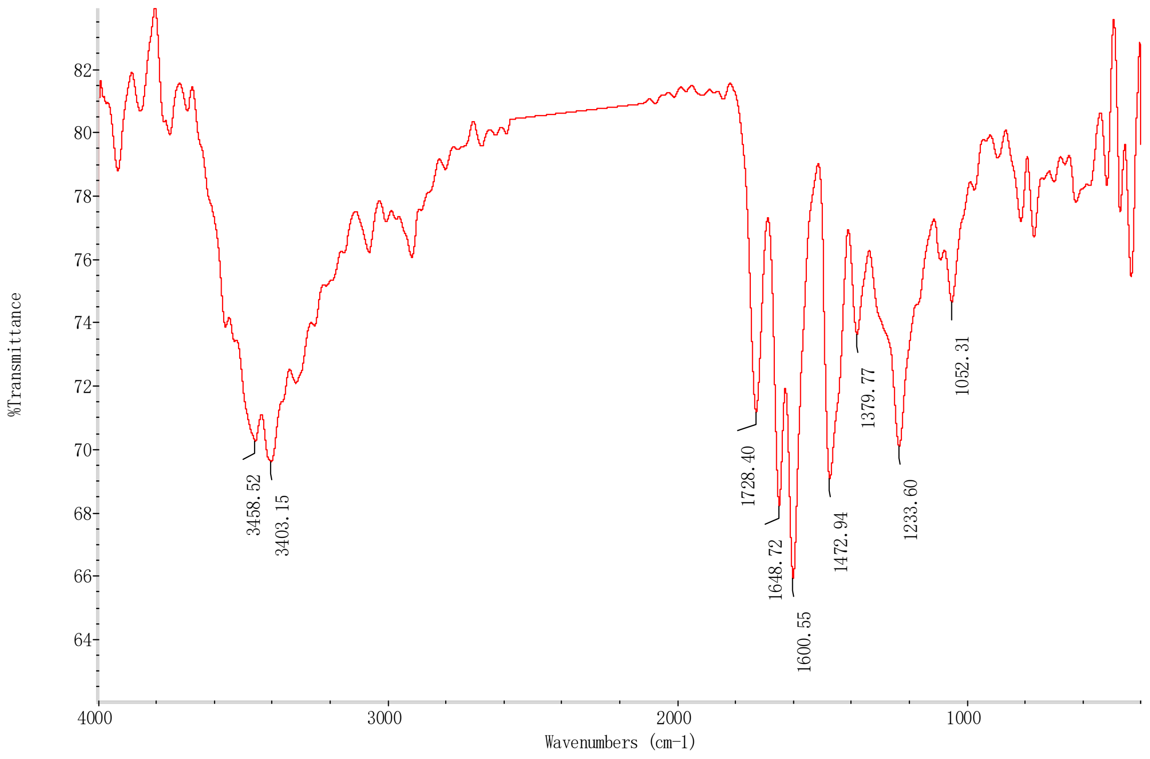

Figure S10. ^{1}H NMR (500 MHz, DMSO-d_6) spectrum of compound **2**

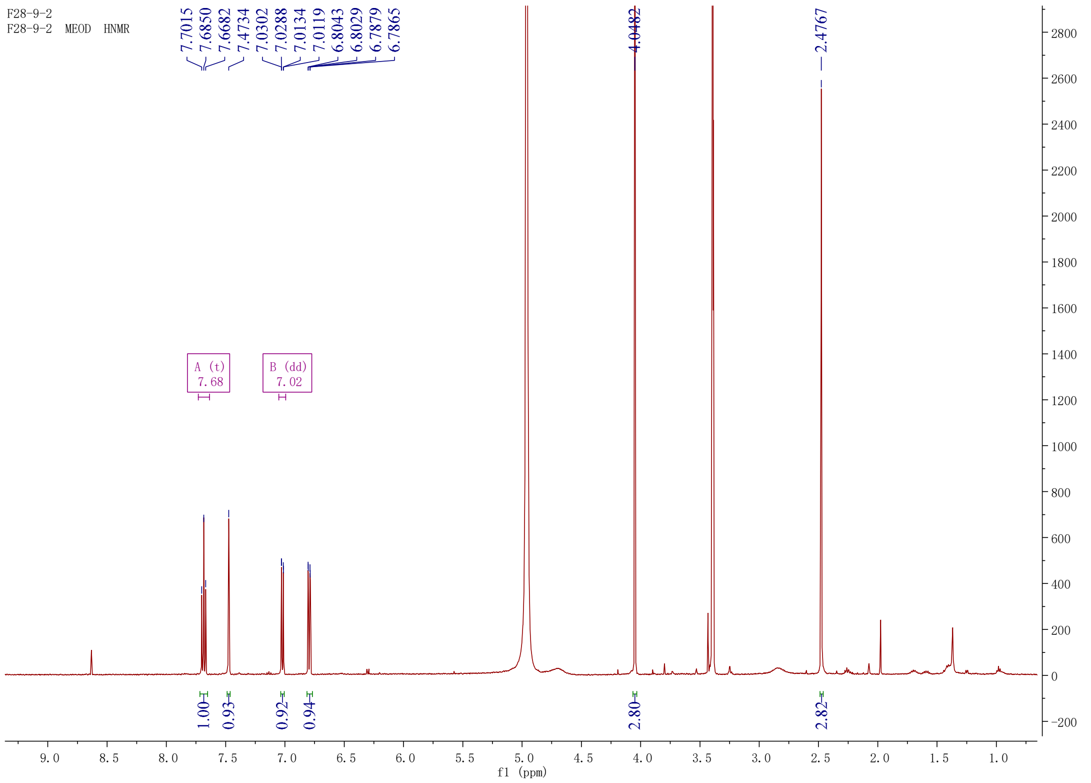

Figure S11. DEPTQ135 (500 MHz, DMSO-d_6) spectrum of compound **2**

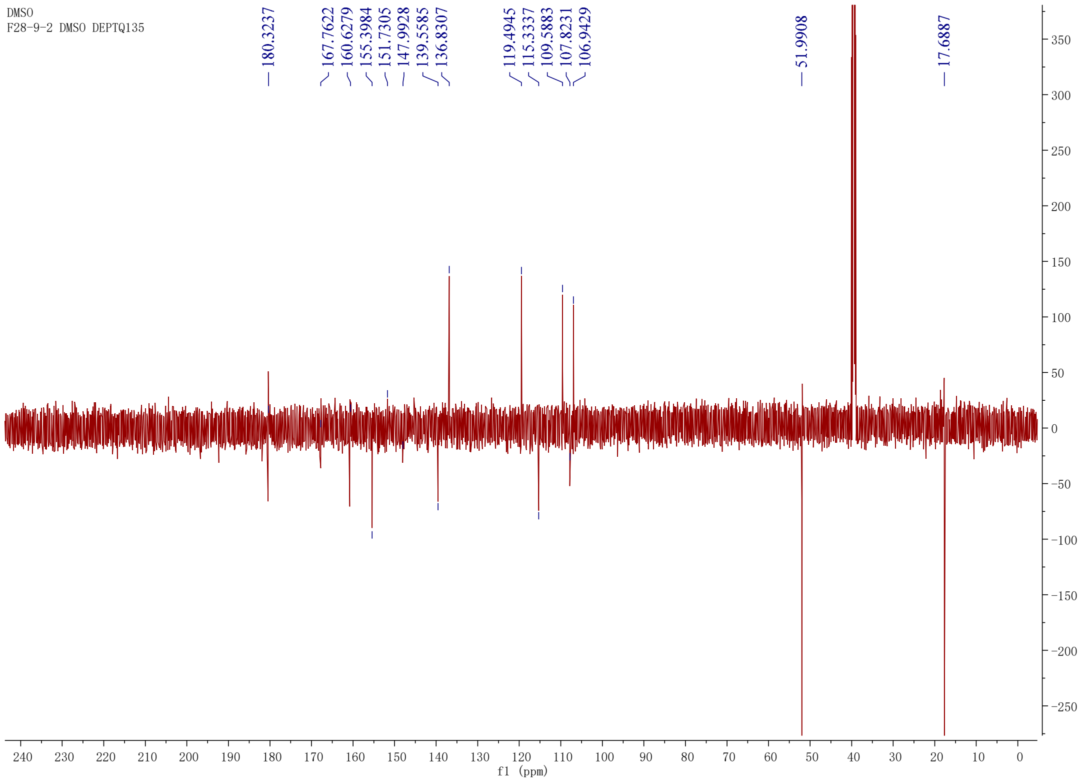

Figure S12. HSQC spectrum of compound **2**

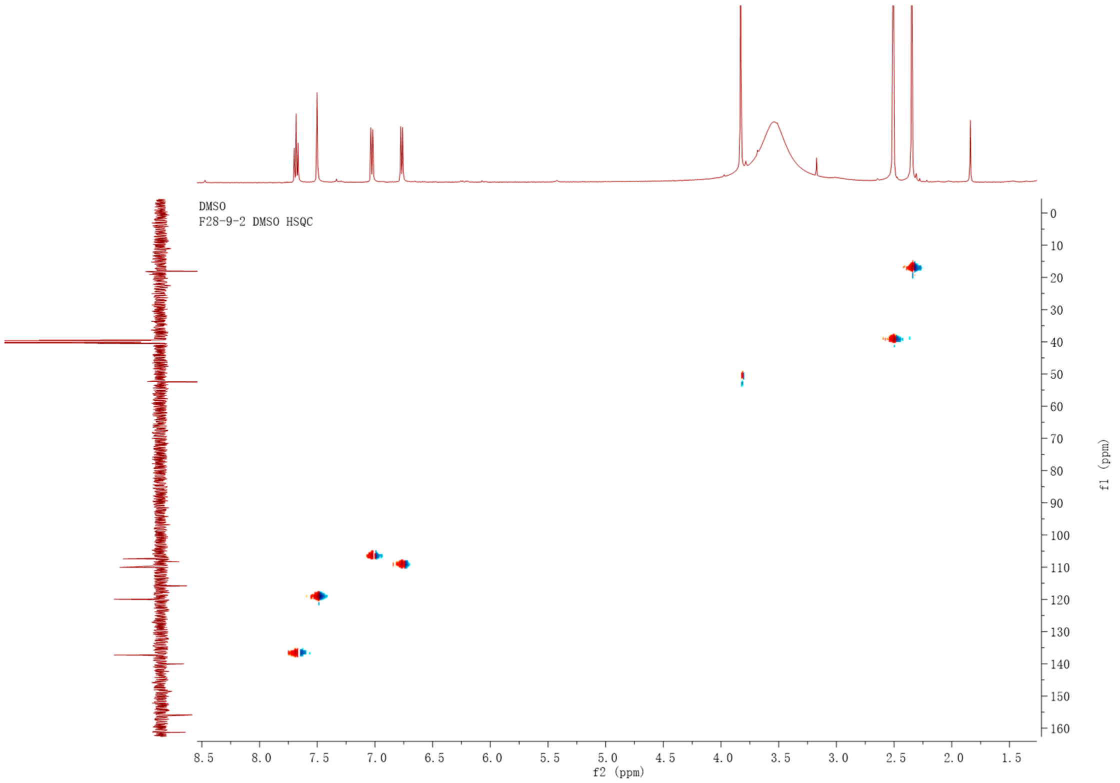

Figure S13. HMBC spectrum of compound **2**

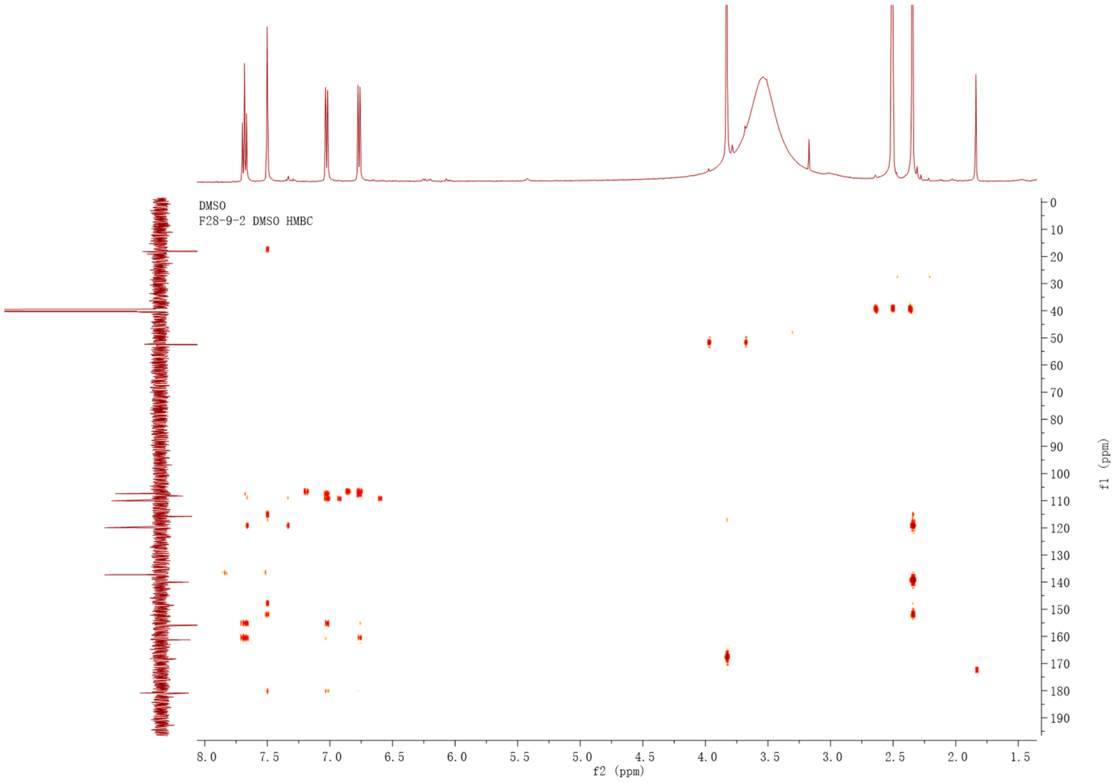

Figure S14. NOESY spectrum of compound **2**

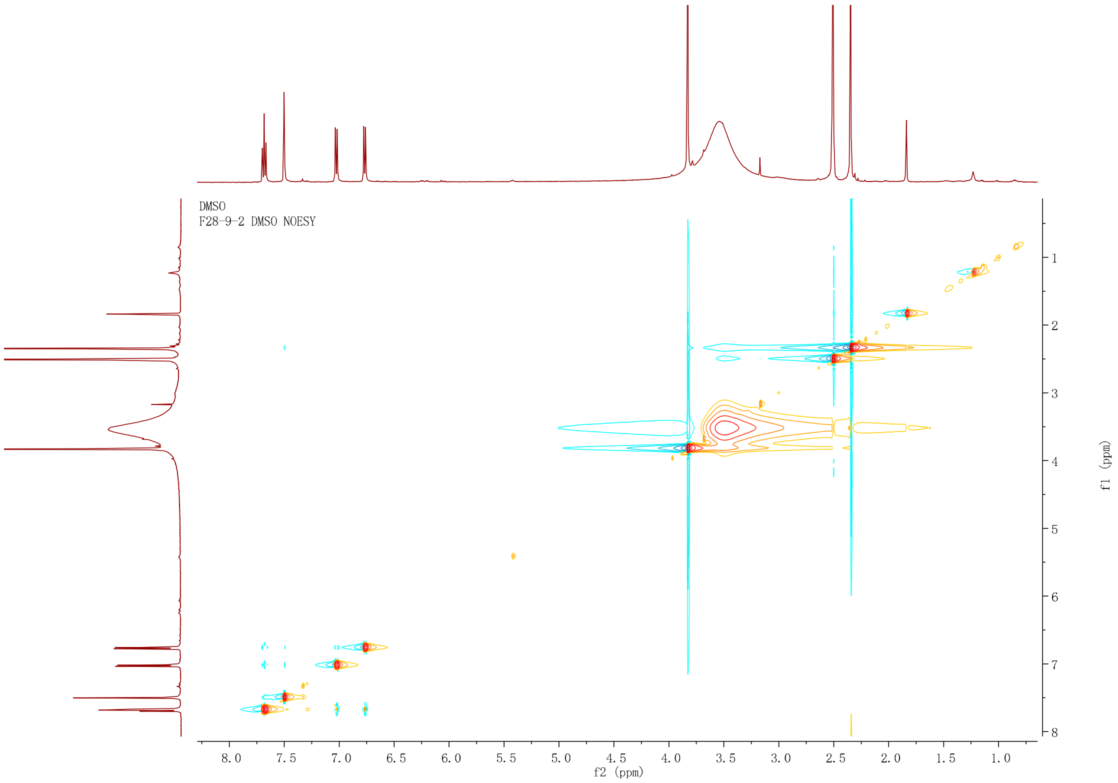

Figure S15. The HRESIMS spectrum of the new compound **2**

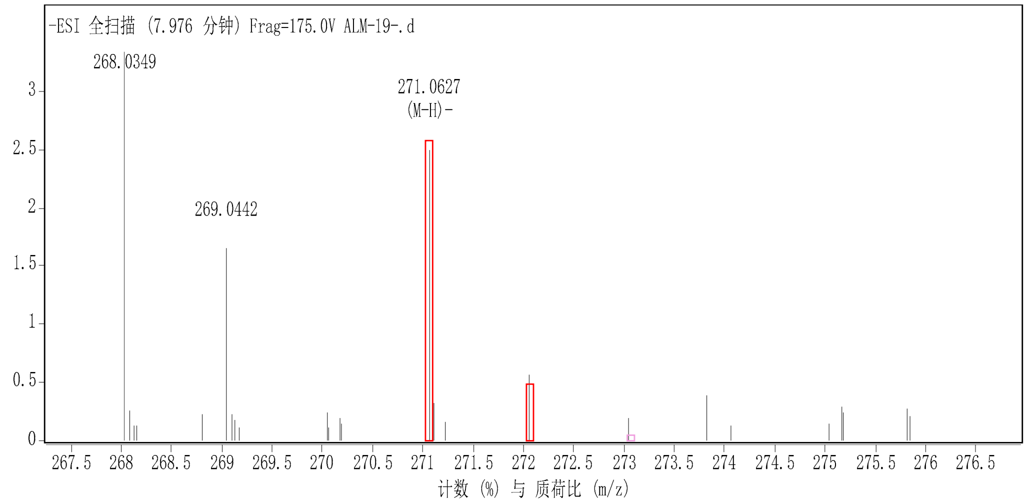

Figure S16. UV spectrum of compound **2**

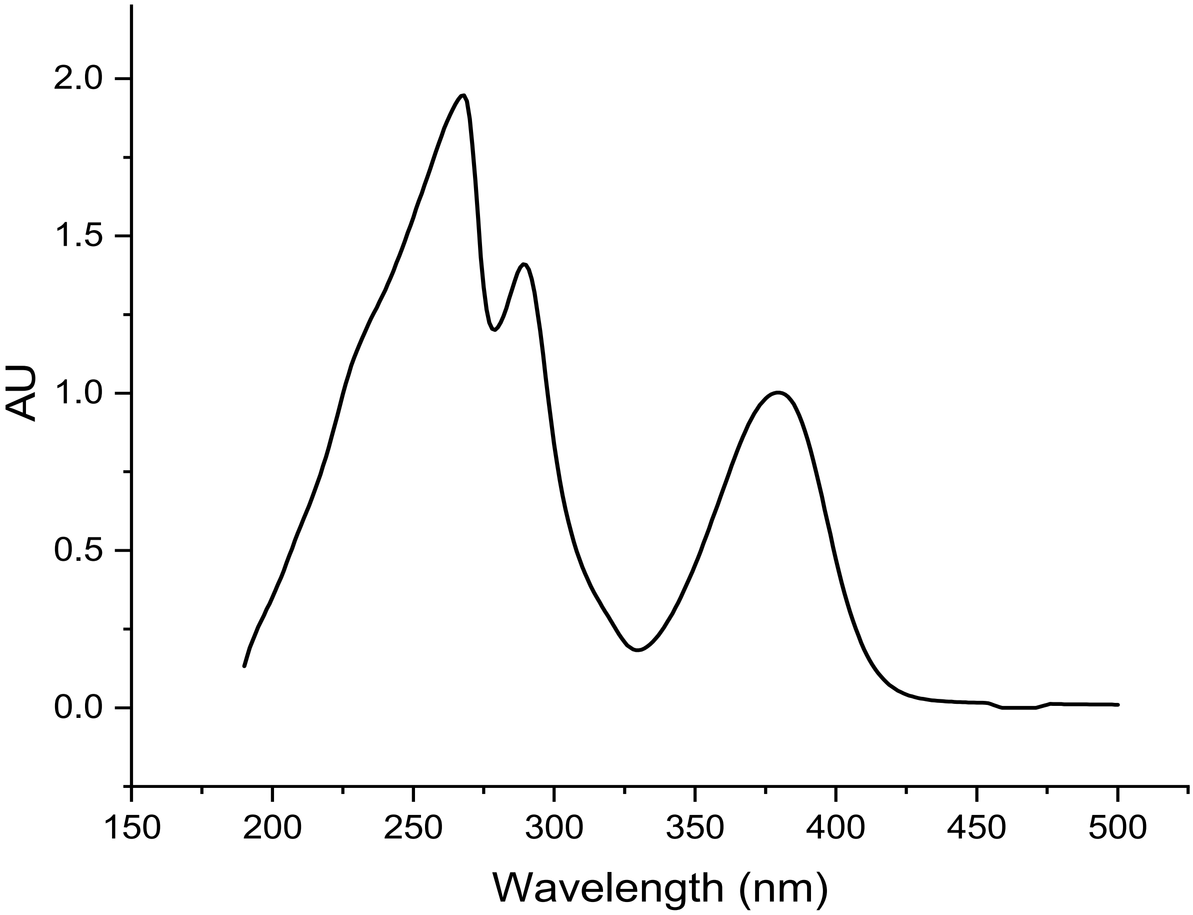

Figure S17. IR spectrum of compound **2**

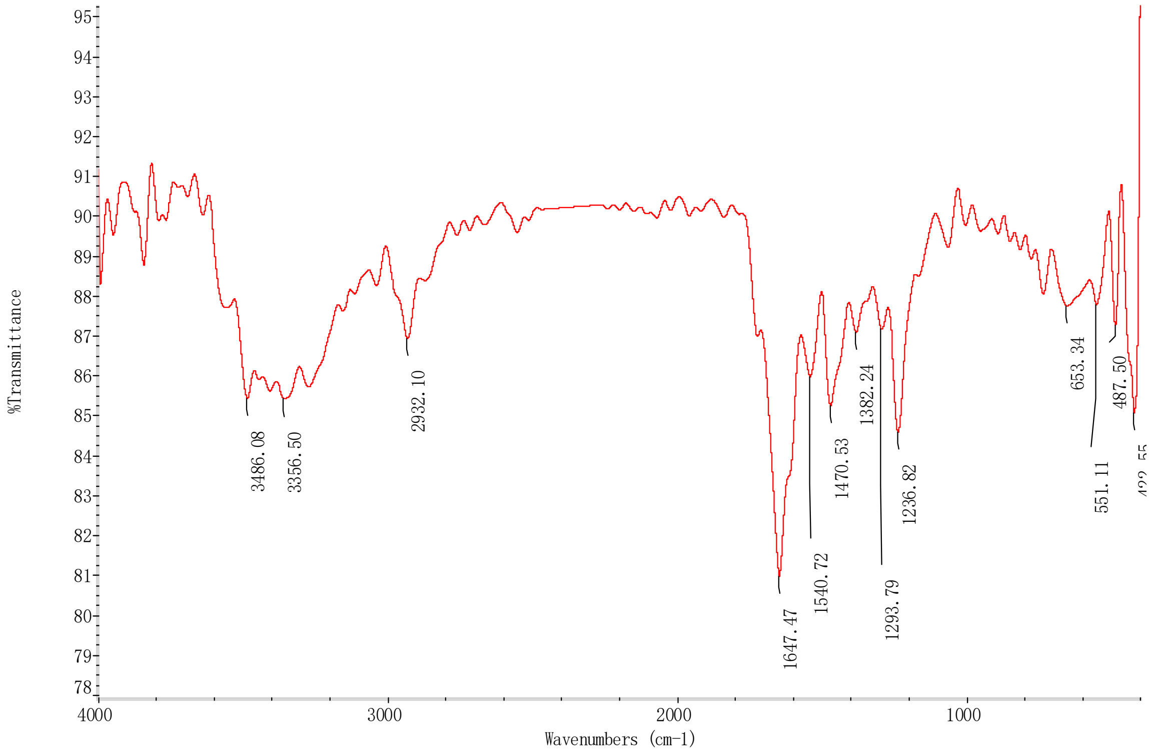

Part Ⅱ

The details of ECD and NMR calculations for 1
The ITS sequence of *Penicillium* sp. F28
Figure S18. Phylogenetic tree of *Penicillium* sp. F28
Isolation procedures of 3–8
Figure S19–35. NMR data of known compounds **3**–**8**
References

The details of ECD and NMR calculations for 1

The conformer rotamer ensemble sampling tool (crest) [1, 2] was utilized to generate candidate conformers and DFT calculations were performed using the Gaussian 16 program [3]. The conformers within an energy window of 10 kcal/mol were optimized at B3LYP/6-31G(d) level of theory with Grimme's D3 dispersion correction ("EmpiricalDispersion=GD3" key words in input files). Frequency analysis of all optimized conformations was undertaken at the same level of theory to ensure they were true local minima on the potential energy surface. Then, energies of all optimized conformations were evaluated by M062X/6-311+G (2d, p) with D3 dispersion correction. Gibbs free energies of each conformers were calculated by adding "Thermal correction to Gibbs Free Energy" obtained by frequency analysis to electronic energies obtained at M062X/6-311+G (2d, p). Room-temperature (298.15 K) equilibrium populations were calculated according to Boltzmann distribution law. Those conformers accounting for over 1% population were subjected to subsequent calculations.

NMR shielding constants were calculated with the GIAO method at mPW1PW91-SCRF/6-31+G (d, p) level with IEFPCM solvent model in chloroform solvent. The shielding constants obtained were converted into chemical shifts by referencing to TMS at 0 ppm ($\delta_{cal} = \sigma_{TMS} - \sigma_{cal}$), where the σ_{TMS} was the shielding constant of TMS calculated at the same level [4]. The DP4+ probabilities of each possible candidate were calculated with the EXCEL spreadsheet provided by Sarotti *et al* [5]. For each possible candidate, the parameters a and b of the linear regression $\delta_{cal} = a\delta_{exp} + b$; the correlation coefficient, R^2; the mean absolute error (MAE) defined as $\Sigma n\ |\delta_{cal} - \delta_{exp}|/n$; the corrected mean absolute error, CMAE, defined as $\Sigma n\ |\delta_{corr} - \delta_{exp}|/n$, where $\delta_{corr} = (\delta_{cal} - b)/a$, were calculated.

Time-dependent density-functional theory (TDDFT) ECD calculations were run at CAM-B3LYP/6-311G(d) level of theory in MeOH with IEFPCM solvent model, respectively. For each conformer, 36 excited states were calculated [6]. The calculated ECD curves were generated using Multiwfn 3.6 software [7].

Table S1. Conformational analysis of the B3LYP/6-31G(d) optimized conformers of (1*S*,2*S*)-**1** in the gas phase (T=298.15 K)

Conformer	E[a] (Hartree)	C[b] (Hartree)	G[c] (kcal/mol)	ΔG[d] (kcal/mol)	Population[e]
(1S,2S)-1-1	-1069.412824	0.226173	-670914.623258	0.0	97.25%
(1S,2S)-1-2	-1069.409446	0.226714	-670912.16438	2.458878	1.53%
(1S,2S)-1-3	-1069.408267	0.225749	-670912.029756	2.593501	1.22%

[a] Electronic energy obtained at M062X/6-311+G (2d, p) level of theory; [b] Thermal correction to Gibbs free energy obtained at B3LYP/6-31G(d) level of theory; [c] Gibbs free energy (E + C); [d] The relative Gibbs free energy; [e] The Boltzmann distribution of each conformer.

Table S2. Atomic coordinates (Å) of (1*S*,2*S*)-1-1 obtained at the B3LYP/6-31G(d) level of theory in the gas phase.

C	-4.648388	-0.013307	0.003671	O	-3.598225	-1.828625	-1.094164

C	-4.546012	1.211795	0.661800	O	3.341715	-1.460732	0.591174
C	-3.315733	1.834216	0.874035	O	1.206106	-1.945126	1.093862
C	-2.174363	1.193422	0.405530	C	1.680214	-2.787538	2.160669
C	-2.226858	-0.041178	-0.264812	H	-5.608192	-0.491297	-0.157608
C	-3.495617	-0.650674	-0.464152	H	-5.449981	1.696135	1.019455
O	-0.972847	1.825488	0.622758	H	-3.228476	2.786335	1.383984
C	0.175131	1.246349	0.198800	H	1.291987	2.821537	1.212190
C	0.212198	0.047021	-0.451585	H	2.121505	1.075459	-2.097390
C	-1.000999	-0.682653	-0.728905	H	1.456802	-1.218220	-1.631904
C	1.376500	2.002743	0.504048	H	3.691057	3.231251	0.862896
C	2.536925	1.699239	-0.112314	H	4.583273	1.730301	0.499757
C	2.533316	0.609886	-1.189439	H	4.212565	2.841856	-0.800176
C	1.566437	-0.523462	-0.791013	H	4.087188	-0.453174	-0.824727
O	-1.002799	-1.783955	-1.324773	H	-2.669319	-2.102489	-1.336407
C	2.146698	-1.342642	0.364754	H	2.247349	-2.197638	2.884575
C	3.818178	2.425688	0.134480	H	0.783488	-3.204841	2.617006
O	3.818107	0.154295	-1.542250	H	2.318554	-3.579393	1.762086

Table S3. Atomic coordinates (Å) of (1*S*,2*S*)-1-2 obtained at the B3LYP/6-31G(d) level of theory in the gas phase.

C	4.505071	0.357833	0.209019	O	3.134950	2.285726	0.191517
C	4.627293	-1.029657	0.139597	O	-2.256887	1.848800	1.222534
C	3.517202	-1.860015	-0.011316	O	-2.163249	2.475142	-0.942121
C	2.265111	-1.260825	-0.090763	C	-2.295498	3.843929	-0.520583
C	2.090004	0.132020	-0.024407	H	5.372521	0.997658	0.326374
C	3.242748	0.951648	0.126910	H	5.614939	-1.476632	0.205467
O	1.184435	-2.093699	-0.237426	H	3.602766	-2.938844	-0.064720
C	-0.062582	-1.572422	-0.311290	H	-0.792140	-3.592866	-0.643775
C	-0.323473	-0.232832	-0.265365	H	-3.751773	-0.580007	-0.170836
C	0.755913	0.713360	-0.122182	H	-1.970107	0.187160	-1.549237
C	-1.104042	-2.573042	-0.439223	H	-4.271397	-2.975466	-0.980812
C	-2.393177	-2.248610	-0.232268	H	-3.157358	-4.256674	-0.446640
C	-2.736680	-0.826083	0.183965	H	-4.006506	-3.236374	0.741845
C	-1.764911	0.185685	-0.469914	H	-2.634971	0.120381	1.864686
O	0.568421	1.953642	-0.082880	H	2.163511	2.495278	0.111782
C	-2.057155	1.585456	0.047581	H	-2.380632	4.423753	-1.439272
C	-3.509357	-3.242743	-0.236730	H	-3.182715	3.970378	0.104051
O	-2.685178	-0.821381	1.598570	H	-1.404879	4.134196	0.041349

Table S4. Atomic coordinates (Å) of (1*S*,2*S*)-1-3 obtained at the B3LYP/6-31G(d) level of theory in the gas phase.

C	-4.760511	-0.325907	0.109432	O	-3.587309	-2.187621	-0.763994
C	-4.741496	0.974448	0.612834	O	1.417907	-1.266372	1.627635
C	-3.556535	1.699765	0.738638	O	3.248527	-1.669009	0.371106
C	-2.374187	1.085911	0.341241	C	3.839809	-2.327359	1.509259
C	-2.343547	-0.220597	-0.176656	H	-5.685293	-0.884493	0.017641
C	-3.566769	-0.936288	-0.285496	H	-5.676186	1.436036	0.917793

O	-1.217691	1.817227	0.475299	H	-3.534257	2.709090	1.132214
C	-0.035473	1.275251	0.101479	H	0.973838	3.009003	0.953555
C	0.078068	0.024334	-0.430580	H	1.918270	0.993801	-2.145079
C	-1.077797	-0.825682	-0.574912	H	1.419767	-1.277728	-1.473959
C	1.114961	2.129779	0.332381	H	4.353532	2.056015	0.330364
C	2.299949	1.832382	-0.237323	H	3.880420	3.037769	-1.036782
C	2.365244	0.646511	-1.201016	H	3.354875	3.508757	0.602000
C	1.460169	-0.504201	-0.700855	H	3.953646	-0.415821	-0.877248
O	-0.997609	-1.996720	-1.012667	H	-2.640467	-2.428996	-0.966661
C	1.994054	-1.171170	0.573006	H	3.218029	-3.170817	1.815681
C	3.531150	2.662142	-0.067086	H	4.818251	-2.668832	1.172469
O	3.679598	0.253678	-1.528902	H	3.935659	-1.629173	2.344119

Table S5. Key transitions, oscillator strengths, and rotatory strengths in the ECD spectrum of conformer (1*S*,2*S*)-1-1 at the CAM-B3LYP/6-311G(d) level of theory in MeOH with IEFPCM solvent model.

Num[a]	*Transition*[b]	*CI-coeff*[b]	*ΔE (eV)*[d]	*λ (nm)*[e]	*f*[f]	R_{vel}[g]	R_{len}[h]
1	79->80	0.66299	3.9717	312.17	0.3020	-16.44	-14.4998
2	78->80	0.55698	4.4365	279.46	0.0234	-20.746	-22.9268
	79->81	0.38922					
3	76->80	0.63604	4.5695	271.33	0.0022	-4.4624	-5.6321
4	77->80	-0.23447	4.9858	248.68	0.2352	-32.3689	-33.3823
	78->80	-0.34826					
	79->81	0.44938					
5	77->80	0.63359	5.2637	235.55	0.5021	-29.5837	-31.1429
	79->81	0.23432					
6	78->81	0.51567	5.5045	225.24	0.0024	31.6011	30.8149
	79->82	0.25189					
7	75->80	0.54236	5.6545	219.27	0.0079	26.9863	25.4418
8	74->82	0.24022	5.7228	216.65	0.0031	32.1155	30.5142
	75->80	0.2531					
	78->81	0.30703					
	78->82	-0.27566					
	79->82	-0.29984					
9	77->81	0.54179	5.9544	208.22	0.0337	-0.9315	-1.1266
	79->83	0.30813					
10	76->81	0.62684	6.2260	199.14	0.0050	7.5367	9.5948
11	74->80	0.25222	6.3275	195.94	0.2172	30.8104	30.3648
	77->81	-0.25674					
	79->83	0.50626					
12	74->80	0.57215	6.4914	191.00	0.0589	10.2747	10.9364
13	73->82	0.22371	6.5342	189.75	0.0131	-22.9265	-22.8276
	74->82	0.41137					
	79->82	0.32244					
14	73->80	0.59086	6.7117	184.73	0.1110	-20.0567	-20.1723
15	78->83	0.47779	6.9192	179.19	0.0216	17.4728	19.9595

	79->84	0.35455					
16	77->83	0.58264	7.0234	176.53	0.2210	-23.5338	-24.6924
17	69->80	0.31105	7.0981	174.67	0.0058	8.9982	9.301
	70->80	0.4219					
	71->80	-0.32625					
18	78->82	0.34918	7.2088	171.99	0.0348	-10.0493	-8.786
	78->83	-0.31272					
	79->82	-0.22958					
	79->84	0.34259					
19	72->80	0.63388	7.2523	170.96	0.0100	2.6252	2.2834
20	71->80	-0.27846	7.3476	168.74	0.0052	0.4254	0.5728
	75->81	0.34656					
	75->82	0.23532					
21	78->82	0.35017	7.3591	168.48	0.0646	19.4233	20.5574
	79->82	-0.26617					
	79->84	-0.22653					
22	76->83	0.53793	7.4219	167.05	0.0120	-30.2651	-32.6284
23	78->86	-0.25562	7.4600	166.20	0.0111	-14.3249	-14.8665
	79->86	0.50578					
24	71->80	0.2859	7.4687	166.00	0.0135	8.9971	9.599
	75->82	0.23007					
	76->82	-0.244					
	79->86	0.27919					
25	73->81	0.38405	7.5302	164.65	0.3638	-27.522	-28.7048
	74->81	-0.32954					
26	75->81	0.40598	7.6824	161.39	0.0463	12.6725	12.6172
	76->82	0.33757					
27	78->84	-0.23182	7.7202	160.60	0.0292	7.6215	7.8861
	78->85	0.33535					
	79->85	0.43523					
28	75->82	0.30346	7.7934	159.09	0.0365	-40.4739	-45.9734
	76->82	0.31205					
	77->82	0.35312					
29	77->82	0.49701	7.8473	158.00	0.0216	-5.1017	-3.4319
30	75->82	0.2674	7.8494	157.95	0.1346	-58.4751	-58.7328
	76->82	0.25433					
	78->84	0.3944					
31	67->80	0.39487	7.8742	157.46	0.0401	45.8996	46.8734
32	69->80	-0.25945	7.9842	155.29	0.0059	-0.8606	-1.8202
	70->80	0.33394					
	74->81	0.41643					
33	66->80	0.35962	8.0321	154.36	0.0003	1.506	1.7447
34	70->80	-0.23079	8.0867	153.32	0.0090	2.6648	4.0516
	73->81	0.42998					
	74->81	0.27					
35	72->82	0.53896	8.1540	152.05	0.1125	-19.0046	-17.3779
36	65->80	0.26813	8.2504	150.28	0.0016	11.0713	11.1474

[a] Number of the excited states; [b] Only transitions with contribution over 10.0% were listed; [c] Configuration-interaction coefficient; [d] Excitation energy; [e] Wavelength; [f] Oscillator strength; [g] Rotatory strength in velocity form (10^{-40} cgs); [h] Rotatory strength in length form (10^{-40} cgs).

Table S6. Key transitions, oscillator strengths, and rotatory strengths in the ECD spectrum of conformer (1*S*,2*S*)-1-2 at the CAM-B3LYP/6-311G(d) level of theory in MeOH with IEFPCM solvent model.

Num[a]	*Transition*[b]	*CI-coeff*[b]	*ΔE (eV)*[d]	*λ (nm)*[e]	*f*[f]	R_{vel}[g]	R_{len}[h]
1	79->80	0.66746	3.9982	310.10	0.2719	-6.4424	-3.9826
2	78->80	0.55594	4.4846	276.47	0.0550	29.6183	28.8692
	79->81	-0.38447					
3	76->80	0.60628	4.5777	270.85	0.0051	13.3594	15.0844
4	77->80	0.2423	4.9871	248.61	0.2745	6.4103	4.7784
	78->80	0.35695					
	79->81	0.45184					
5	77->80	0.63594	5.2305	237.04	0.5061	-9.3738	-9.8031
	79->81	-0.23026					
6	75->80	0.58109	5.4807	226.22	0.0317	1.4087	3.1369
	76->80	-0.23188					
7	78->81	0.58599	5.6985	217.57	0.0038	-28.8827	-26.7721
8	74->80	0.52006	5.9454	208.54	0.0030	0.492	0.8749
	74->82	0.23899					
	74->83	0.23393					
9	77->81	0.53736	5.9619	207.96	0.0416	3.9639	3.0802
	79->82	0.27882					
10	74->80	-0.29466	6.0732	204.15	0.0018	-4.8445	-4.3962
	74->82	0.27156					
	74->83	0.40367					
11	76->81	0.56433	6.2518	198.32	0.0163	-12.4642	-14.8755
12	73->80	0.28654	6.3567	195.04	0.2609	-40.511	-39.567
	77->81	-0.2727					
	79->82	0.44526					
13	78->82	0.26807	6.4217	193.07	0.0047	-3.951	-2.9112
	78->83	0.2398					
	79->83	0.45973					
14	73->80	0.50835	6.6095	187.59	0.1039	10.5222	11.3088
	77->81	0.26065					
15	77->82	0.25845	6.9807	177.61	0.0715	4.776	4.3894
	78->82	-0.27851					
	78->83	0.26791					
	79->84	0.33926					
16	71->80	-0.24728	7.0482	175.91	0.0889	21.1506	19.8314
	77->82	0.35351					
	77->83	-0.28038					
17	71->80	0.35745	7.1229	174.06	0.1065	-55.5481	-54.4821
	72->80	0.33461					

18	71->80	-0.2335	7.1748	172.80	0.0063	-5.8743	-5.1356
	72->80	0.5641					
19	75->81	0.41692	7.2340	171.39	0.0222	1.3715	1.4587
	79->84	-0.25607					
20	69->80	0.55488	7.2799	170.31	0.0042	-12.8489	-12.085
21	75->81	0.24929	7.3318	169.10	0.1079	-9.566	-9.6955
	76->82	0.23854					
	78->82	0.3254					
	79->84	0.31221					
22	76->82	0.34705	7.3710	168.21	0.0102	-1.9252	-2.009
	76->83	0.40771					
	78->82	-0.22591					
23	76->82	0.37374	7.4624	166.15	0.0203	-36.6199	-38.4158
	76->83	-0.33694					
24	79->86	0.59502	7.4714	165.95	0.0054	6.2129	6.2538
25	73->81	0.3018	7.5153	164.97	0.1144	-21.0892	-21.2071
	78->83	-0.25691					
26	73->81	0.24204	7.5505	164.21	0.1350	25.7729	26.6411
	74->81	-0.27486					
	78->83	0.33505					
27	75->82	0.34966	7.6197	162.72	0.0251	1.3643	0.8156
	75->83	0.43193					
28	67->80	0.31026	7.6584	161.89	0.0303	34.4812	37.2437
	73->81	0.25594					
	74->81	0.34918					
29	67->80	0.43216	7.7005	161.01	0.0297	-4.1618	-4.4302
	74->81	-0.22484					
30	78->85	0.45345	7.7880	159.20	0.0413	6.2294	6.7078
	79->85	0.45202					
31	77->82	0.36685	7.8308	158.33	0.1585	80.8794	79.2261
	77->83	0.23952					
	78->84	0.42213					
32	77->83	0.39051	7.9385	156.18	0.1428	-15.6802	-16.1324
	78->84	-0.35164					
33	66->80	0.26321	7.9790	155.39	0.0055	-0.7647	-1.091
	69->81	0.26611					
	70->80	-0.26123					
34	70->80	0.36993	8.0267	154.46	0.0108	-0.08	-2.0997
	73->81	0.26657					
35	65->80	-0.26637	8.1150	152.78	0.0476	-7.7841	-7.2841
	72->82	0.27691					
	72->83	0.36237					
36	66->80	0.27168	8.1480	152.17	0.0592	14.6094	18.0601
	72->82	0.26783					
	72->83	0.33353					

[a] Number of the excited states; [b] Only transitions with contribution over 10.0% were listed; [c] Configuration-interaction coefficient; [d] Excitation energy; [e] Wavelength; [f] Oscillator

strength; [g] Rotatory strength in velocity form (10^{-40} cgs); [h] Rotatory strength in length form (10^{-40} cgs).

Table S7. Key transitions, oscillator strengths, and rotatory strengths in the ECD spectrum of conformer (1*S*,2*S*)-1-3 at the CAM-B3LYP/6-311G(d) level of theory in MeOH with IEFPCM solvent model.

Num[a]	*Transition*[b]	*CI-coeff*[b]	*ΔE (eV)*[d]	*λ (nm)*[e]	*f*[f]	R_{vel}[g]	R_{len}[h]
1	79->80	0.66175	4.0098	309.21	0.3095	-12.5114	-10.5733
2	78->80	0.56806	4.4663	277.60	0.0496	-27.026	-29.1257
	79->81	0.37778					
3	76->80	0.64091	4.5716	271.20	0.0026	-4.7542	-6.3875
4	77->80	-0.23926	4.9955	248.19	0.2576	-22.5499	-23.0647
	78->80	-0.3338					
	79->81	0.46006					
5	77->80	0.63569	5.2662	235.43	0.4710	-3.7239	-4.4711
	79->81	0.22852					
6	78->81	0.55712	5.6396	219.85	0.0029	26.8269	27.6582
7	75->80	0.57194	5.6843	218.12	0.0068	27.7997	23.6167
	78->81	-0.22548					
8	74->82	0.53252	5.8834	210.74	0.0036	26.7918	26.1741
	78->82	-0.2503					
	79->82	-0.24696					
9	77->81	0.54066	5.9691	207.71	0.0357	-9.3547	-9.3896
	79->83	0.30883					
10	74->82	0.30523	6.0607	204.57	0.0038	-6.7049	-6.1446
	78->82	0.36685					
	79->82	0.44931					
11	74->80	0.28893	6.2353	198.84	0.0036	-4.1704	-3.8995
	76->81	0.57317					
12	74->80	0.55281	6.2791	197.46	0.0454	41.2757	44.778
	76->81	-0.27071					
	79->83	-0.23793					
13	73->80	-0.28906	6.3526	195.17	0.1970	-2.1359	-3.2524
	74->80	0.26955					
	77->81	-0.24904					
	79->83	0.46874					
14	73->80	0.52553	6.6421	186.66	0.1251	-10.6965	-10.07
	77->81	-0.28323					
15	78->83	0.47257	6.9609	178.11	0.0619	11.6937	13.4846
	79->84	-0.3327					
16	77->83	0.5855	7.0456	175.97	0.2045	-18.0127	-17.0746
17	69->80	0.54515	7.1509	173.38	0.0014	4.6428	5.0612
	70->80	0.26186					
18	71->80	-0.27859	7.1900	172.44	0.0316	4.9234	4.5835
	72->80	0.57341					
19	78->82	0.50194	7.2128	171.90	0.0111	-13.5739	-12.9266

	79->82	-0.38712					
20	78->83	0.33217	7.3187	169.41	0.0158	7.6578	7.5187
	79->84	0.38339					
21	76->83	0.53435	7.4175	167.15	0.0017	11.0391	11.1819
22	71->80	0.24487	7.4557	166.29	0.0661	-28.7545	-30.1667
	75->81	0.32042					
	76->83	-0.22997					
23	78->86	-0.24111	7.4672	166.04	0.0065	-7.4353	-7.8999
	79->86	0.58711					
24	75->82	-0.3964	7.5116	165.06	0.0062	-0.7159	-0.5304
	76->82	0.45077					
25	70->80	0.24224	7.5375	164.49	0.3271	-51.3069	-53.8919
	73->81	0.45009					
26	71->80	-0.31611	7.6205	162.70	0.0237	9.4224	8.8133
	75->81	0.39777					
27	74->81	0.4425	7.7431	160.12	0.0224	-4.7916	-3.7998
	75->82	-0.28317					
	76->82	-0.35985					
28	74->81	-0.33149	7.7471	160.04	0.0017	2.4103	2.052
	75->82	-0.25277					
	77->82	0.37587					
29	74->81	0.23656	7.7567	159.84	0.0170	-15.5845	-16.3098
	76->82	0.29553					
	77->82	0.42269					
30	77->82	0.27867	7.8319	158.31	0.1347	-37.753	-40.2897
	78->84	0.43029					
	79->85	0.25733					
31	68->80	0.41116	7.8646	157.65	0.0067	-12.5347	-12.5091
32	68->80	0.25457	7.8875	157.19	0.1331	30.618	31.896
	78->84	-0.27889					
	78->85	0.31794					
33	67->80	0.40111	7.9531	155.89	0.0126	-5.1937	-4.7379
34	69->80	-0.24675	8.0191	154.61	0.0068	1.712	1.0988
	70->80	0.4807					
	73->81	-0.32283					
35	72->82	0.62453	8.1605	151.93	0.0940	-3.4439	-3.7736
36	65->80	0.2868	8.2112	150.99	0.0009	6.9604	7.0906
	66->80	0.2382					
	68->80	0.25114					
	68->81	0.2708					

[a] Number of the excited states; [b] Only transitions with contribution over 10.0% were listed; [c] Configuration-interaction coefficient; [d] Excitation energy; [e] Wavelength; [f] Oscillator strength; [g] Rotatory strength in velocity form (10^{-40} cgs); [h] Rotatory strength in length form (10^{-40} cgs).

The ITS sequence of *Penicillium* sp. F28

>F28_ITS Contig4:26877-27458(+) ITS1-ITS4 582 bp
TCCGTAGGTGAACCTGCGGAAGGATCATTACTGAGTGCGGGCCCTCTGGGTCCA
ACCTCCCACCCGTGTATACCGTACCT
TGTTGCTTCGGCGGGCCCGCCAGTCTGGCCGCCGGGGGGCATCTGCCCCCGGGCC
CGCGCCCGCCGGAGACATCATTGAA
CGCTGTCTGAAGATTGCAGTCTGAGCGATAAGCACAAATTAGTTAAAACTTTCAA
CAACGGATCTCTTGGTTCCGGCATC
GATGAAGAACGCAGCGAAATGCGATAAGTAATGTGAATTGCAGAATTCAGTGA
ATCATCGAGTCTTTGAACGCACATTGC
GCCCCCTGGTATTCCGGGGGGCATGCCTGTCCGAGCGTCATTGCTGCCCTCAAGC
CCGGCTTGTGTGTTGGGCGCCGTCC
CCCCGGGGACGGGCCCGAAAGGCAGCGGCGGCACCGCGTCCGGTCCTCGAGCG
TATGGGGCTCTGTCACCCGCTCCGCAG
GCCCGGCCGGCGCCAGCCGACCCCCTCAACCCCTTTTTTTCAGGTTGACCTCGGA
TCAGGTAGGGATACCCGCTGAACTT
AAGCATATCAATAAGCGGAGGA

Phylogenetic tree of *Penicillium* sp. F28

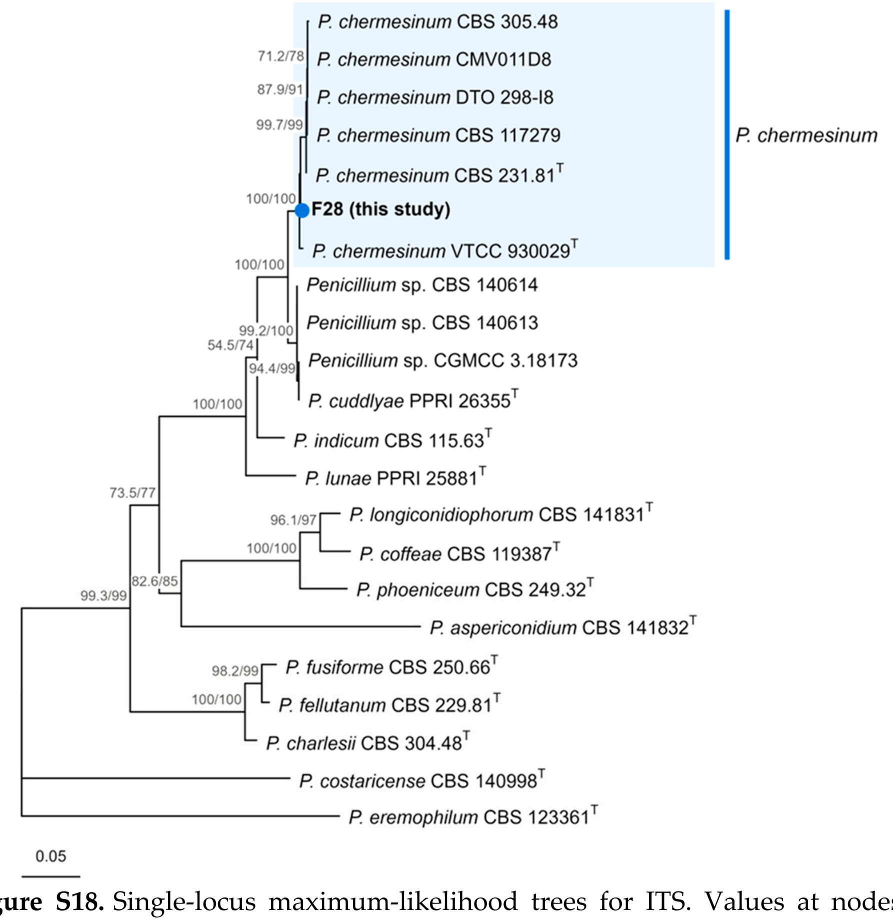

Figure S18. Single-locus maximum-likelihood trees for ITS. Values at nodes are SH-aLRT (%)/UFBoot (%), shown where SH-aLRT ≥ 80 and UFBoot ≥ 95. Blue dots mark strain F28; where *P. chermesinum* is monophyletic, the clade is shaded and the blue vertical bar marks it (in the

ITS tree it is not monophyletic). T= ex-type strain (VTCC 930029 is the ex-type of *P. vietnamense*, now treated under *P. chermesinum*); scale bars indicate substitutions per site.

Isolation procedures

The fungal culture was exhaustively extracted using EtOAc to obtain a crude extract (90.0 g), which was fractionated via silica gel vacuum liquid chromatography with the elution gradient of chloroform ($CHCl_3$)/methanol (MeOH) (100:1, 50:1, 25:1, 10:1, 5:1, 2:1, 1:1, 0:1) to yield five fractions [Fractions (Frs.) 1–5].

Fr. 2 was purified via CC over RP-C18 eluting with a MeOH–H_2O gradient (from 5:5 to 1:0) to obtain four subfractions (Fr. 2-1 to 2-4). Fr. 2-1 was purified by Sephadex LH-20 (MeOH) and then further purified by using semi-preparative HPLC (60% MeOH–H_2O) to obtain **3** (2.1 mg, t_R 50.0 min).

Fr. 5 was purified via RP-C18 eluting with a MeOH–H_2O gradient (0:1, 1:9, 2:8, 3:7, 4:6, 5:5, 6:4, 7:3, 8:2, 9:1, 1:0) to obtain ten subfractions (Fr.5-1 to 5-10). Fr. 5-3 was purified by Sephadex LH-20 (MeOH) and then further purified by using semi-preparative HPLC (20% MeOH–H_2O) to obtain **4** (2.0 mg, t_R 9.0 min). Fr. 5-4 was purified by Sephadex LH-20 (MeOH) and then further purified by using semi-preparative HPLC (30% MeOH–H_2O) to obtain **5** (2.8 mg, t_R 10.0 min), (28% MeOH–H_2O) to obtain **6** (1.8 mg, t_R 11.0 min).

Fr. 4 was purified via RP-C18 eluting with a MeOH–H_2O gradient (0:1, 1:9, 2:8, 3:7, 4:6, 5:5, 6:4, 7:3, 8:2, 9:1, 1:0) to obtain ten subfractions (Fr. 4-1 to 4-10). Fr. 4-7 was purified by Sephadex LH-20 (MeOH) to obtain **7** (1.5 mg).

Fr. 3 was purified via RP-C18 eluting with a MeOH–H_2O gradient (0:1, 1:9, 2:8, 3:7, 4:6, 5:5, 6:4, 7:3, 8:2, 9:1, 1:0) to obtain ten subfractions (Fr. 3-1 to 3-10). Fr. 3-3 was purified by Sephadex LH-20 (MeOH) and then further purified by using semi-preparative HPLC (30% MeOH–H_2O) to obtain **8** (2.2 mg, t_R 10.0 min).

Figure S19. ^{1}H NMR (500 MHz, methanol-d_4) spectrum of compound **3**

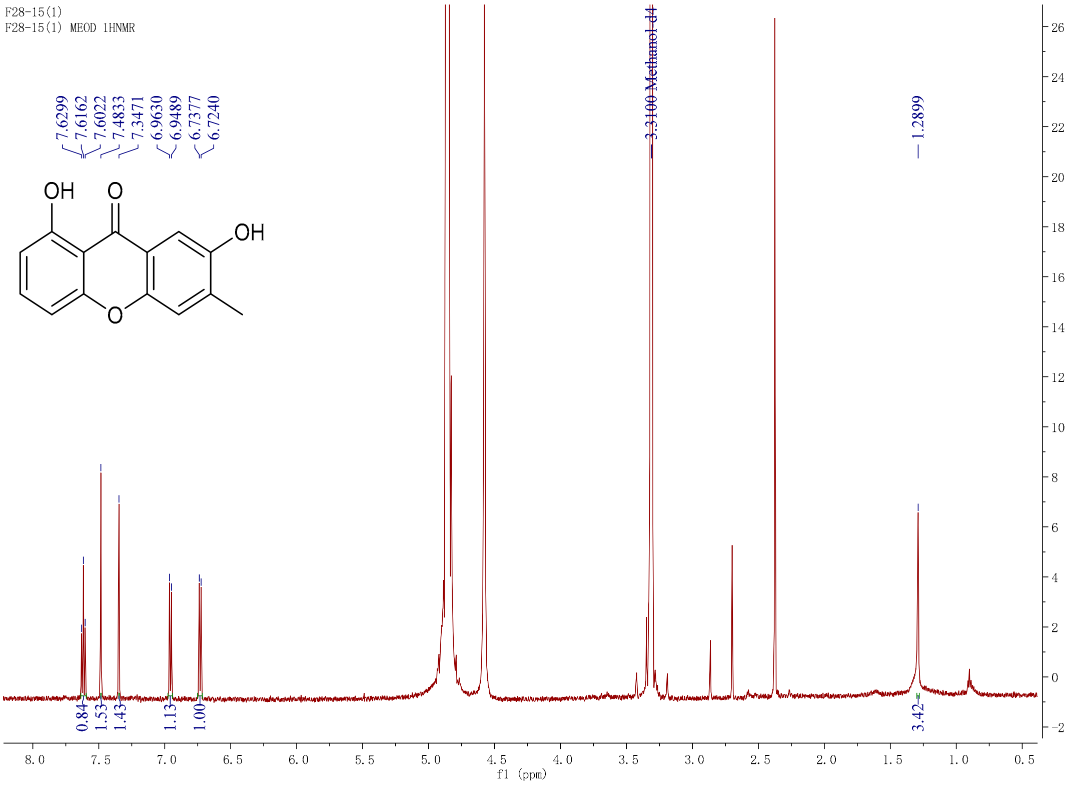

Figure S20. ^{13}C NMR (125 MHz, methanol-d_4) spectrum of compound **3**

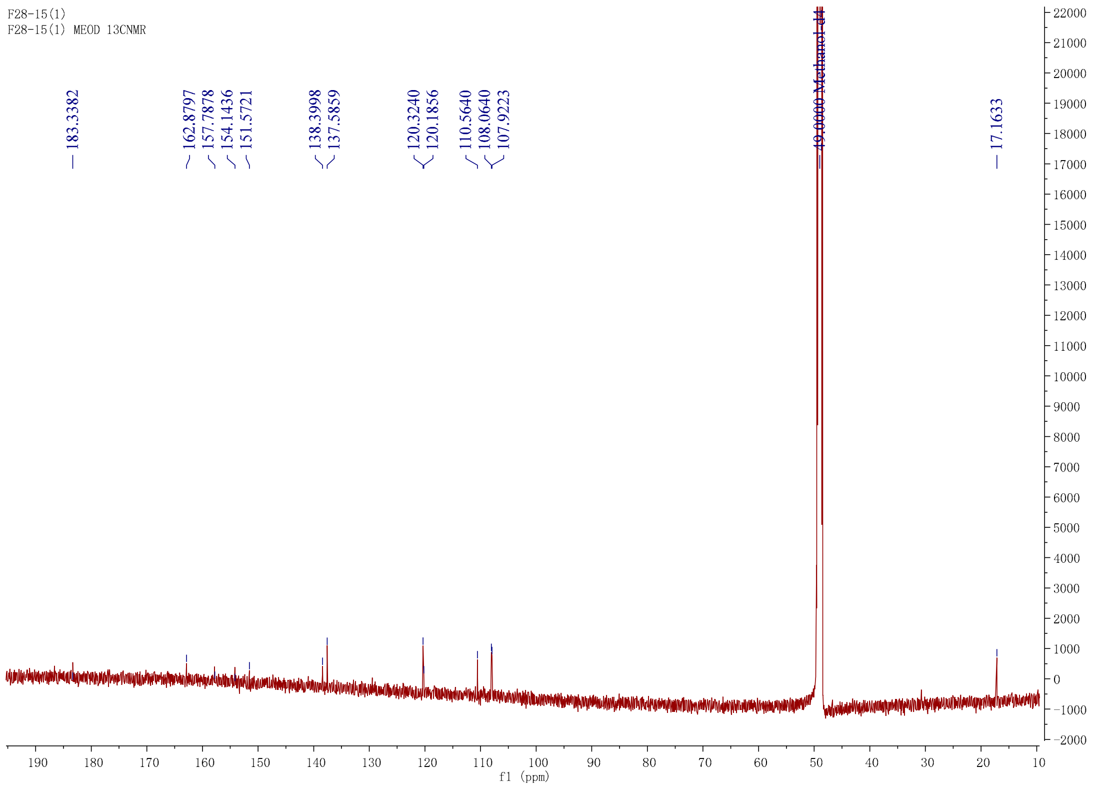

Figure S21. ^{1}H NMR (400 MHz, methanol-d_4) spectrum of compound **4**

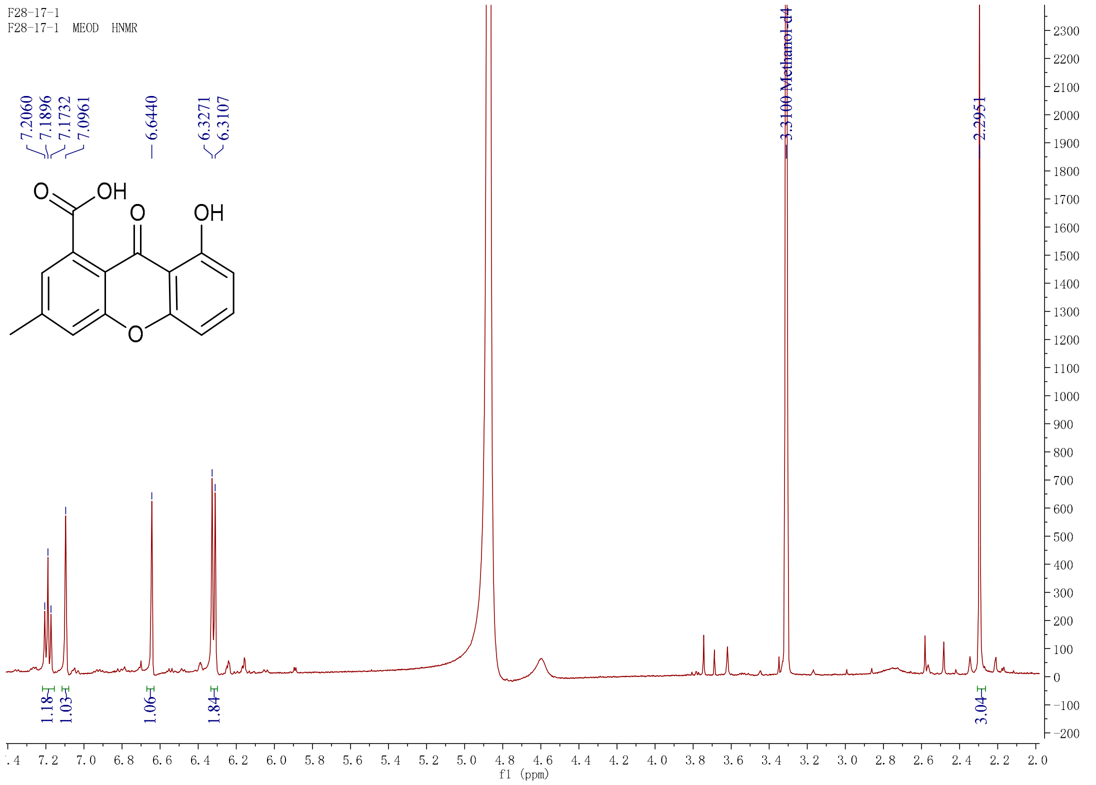

Figure S22. ^{13}C NMR (100 MHz, methanol-d_4) spectrum of compound **4**

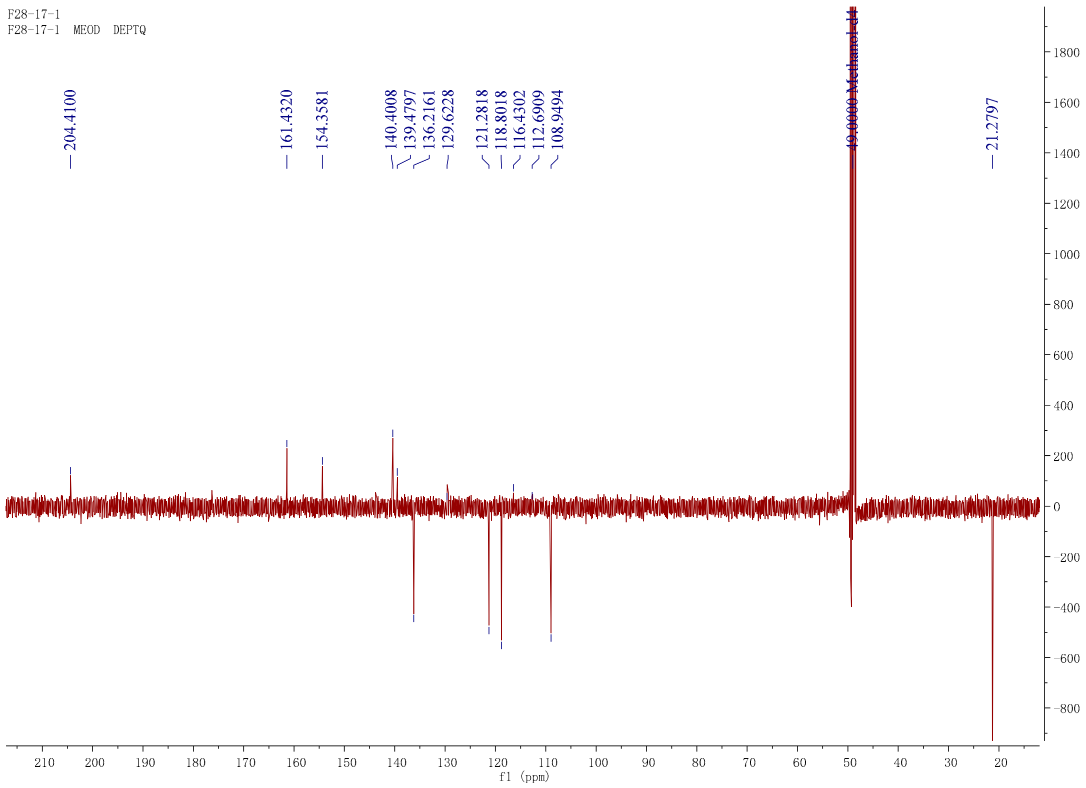

Figure S23. ^{1}H NMR (400 MHz, chloroform -d) spectrum of compound **5**

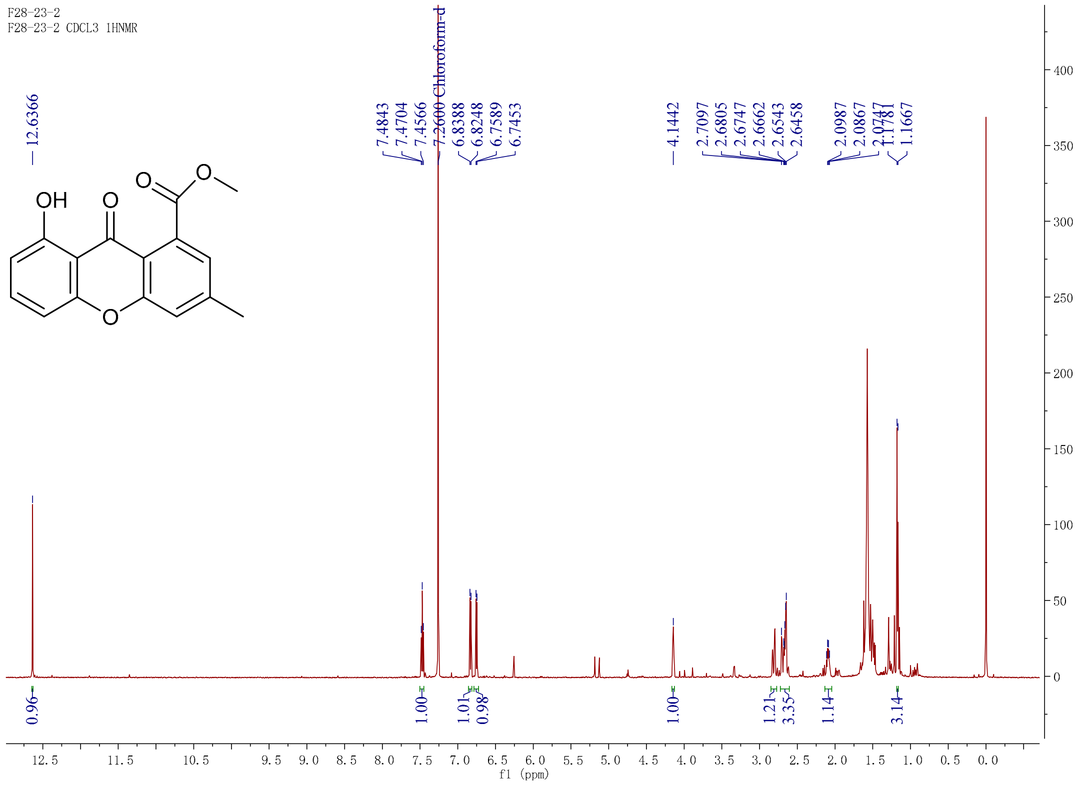

Figure S24. ^{13}C NMR (100 MHz, chloroform -*d*) spectrum of compound **5**

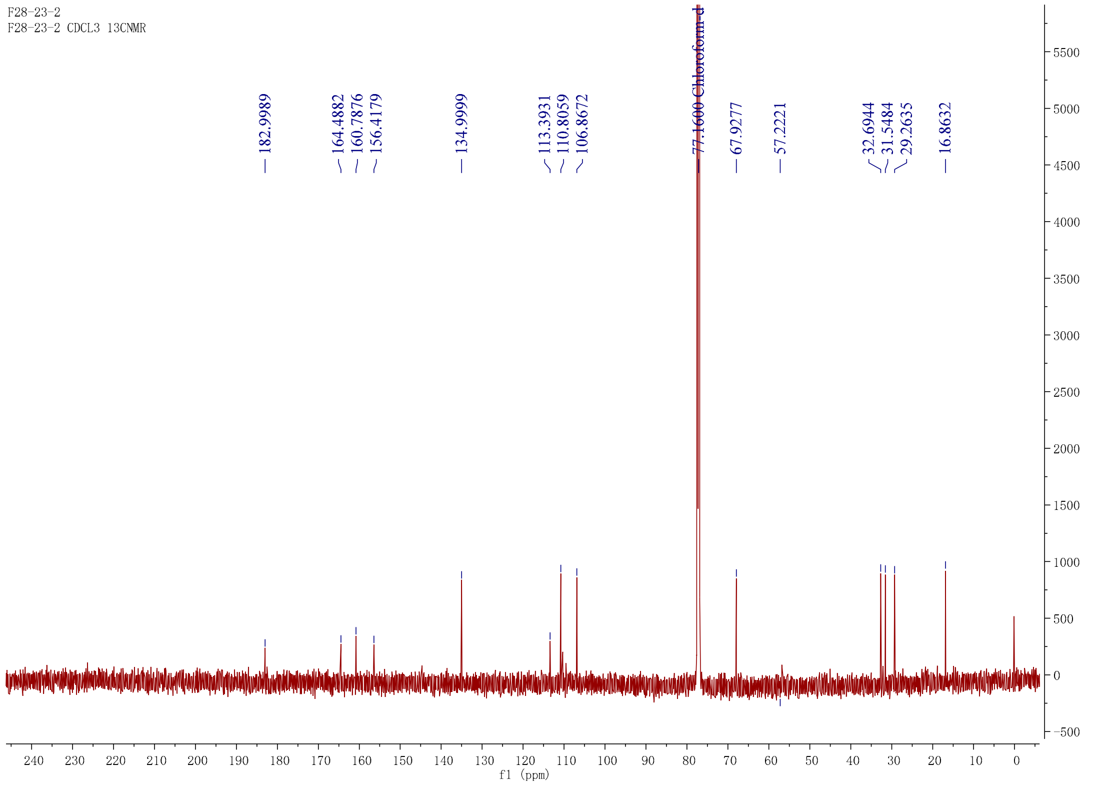

Figure S25. DEPT135 spectrum of compound **5**

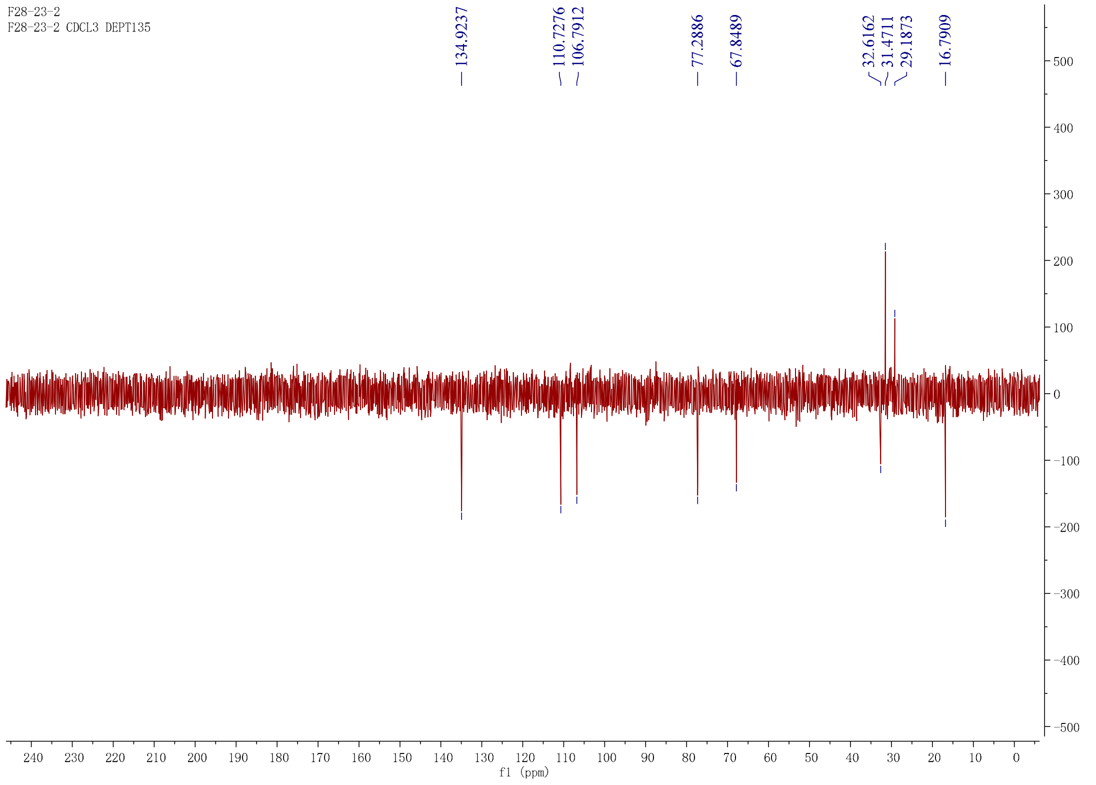

Figure S26. HSQC spectrum of compound **5**

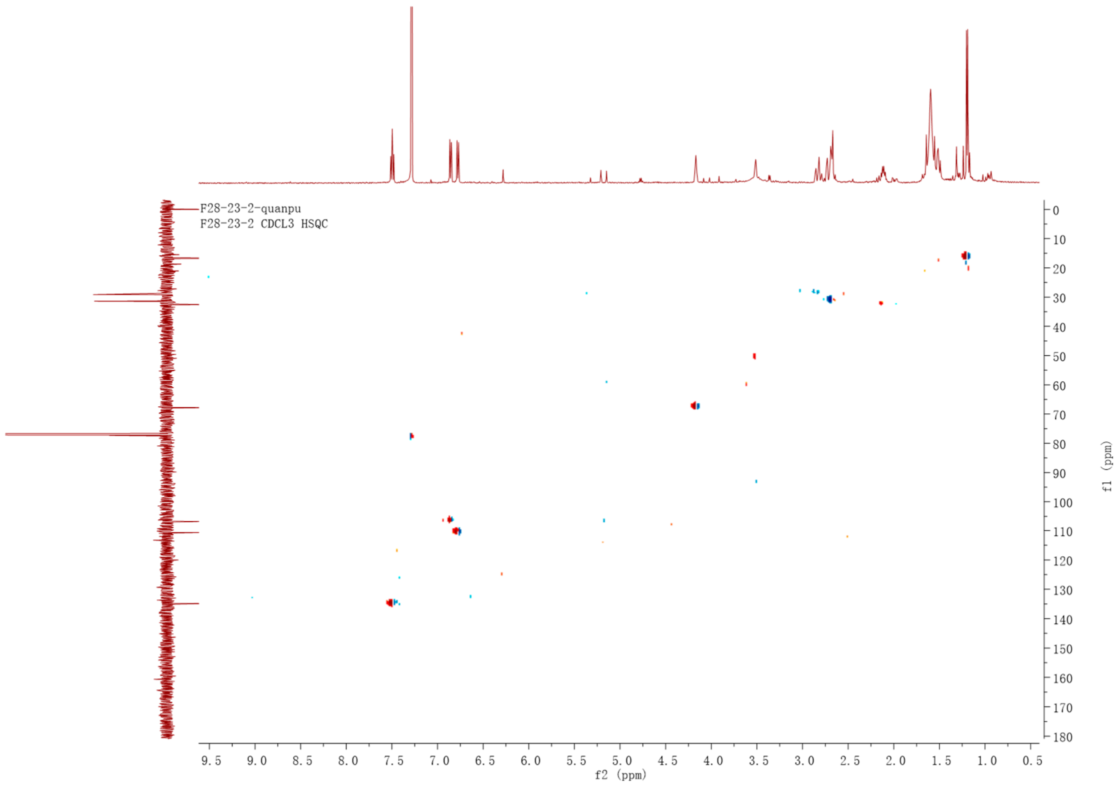

Figure S27. HMBC spectrum of compound **5**

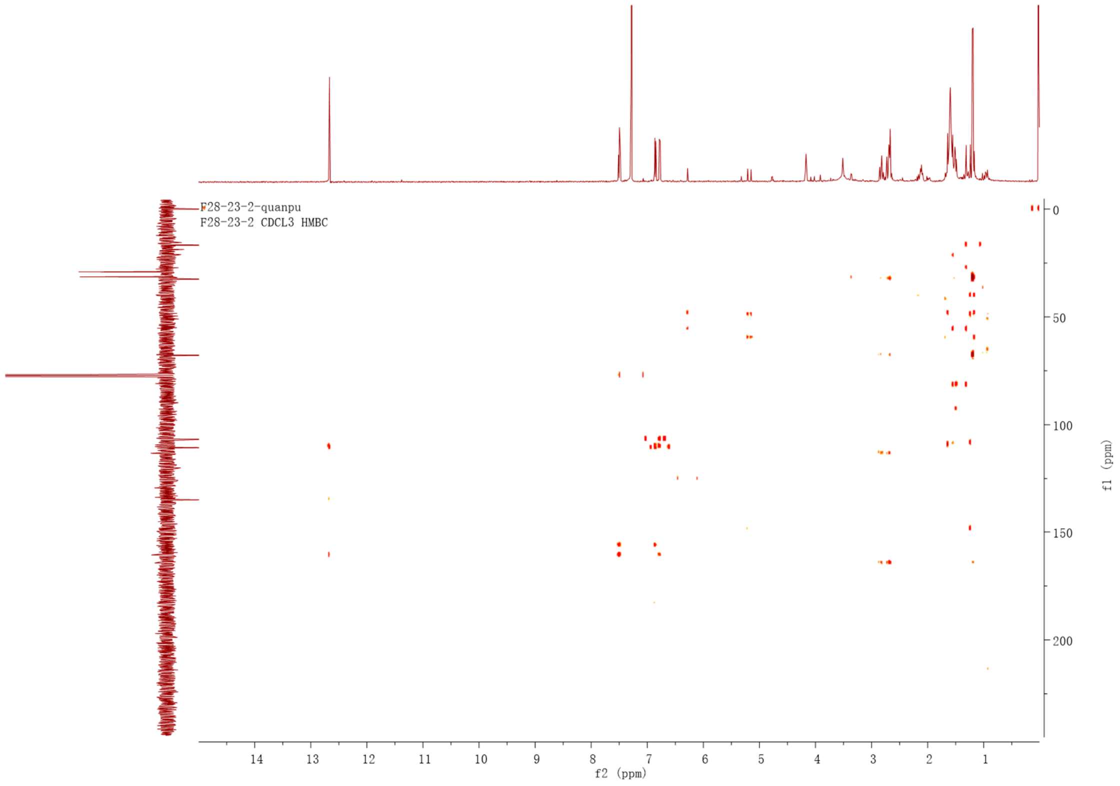

Figure S28. ^{1}H-^{1}H COSY spectrum of compound **5**

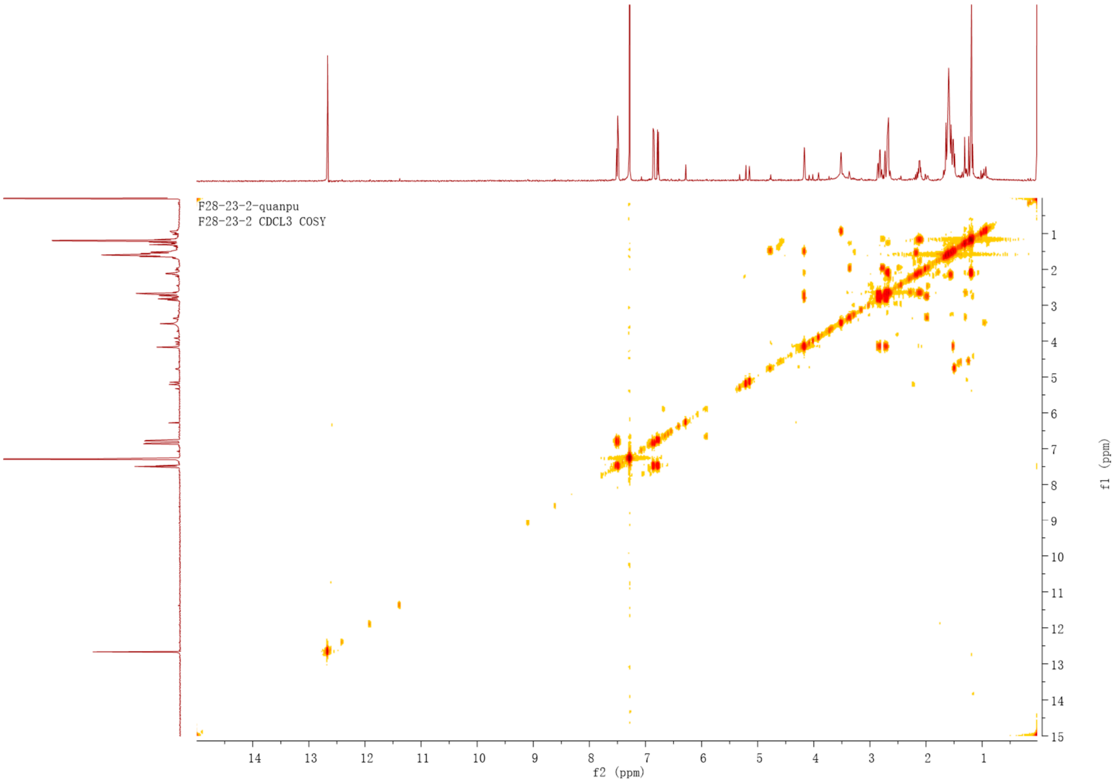

Figure S29. NOESY spectrum of compound **5**

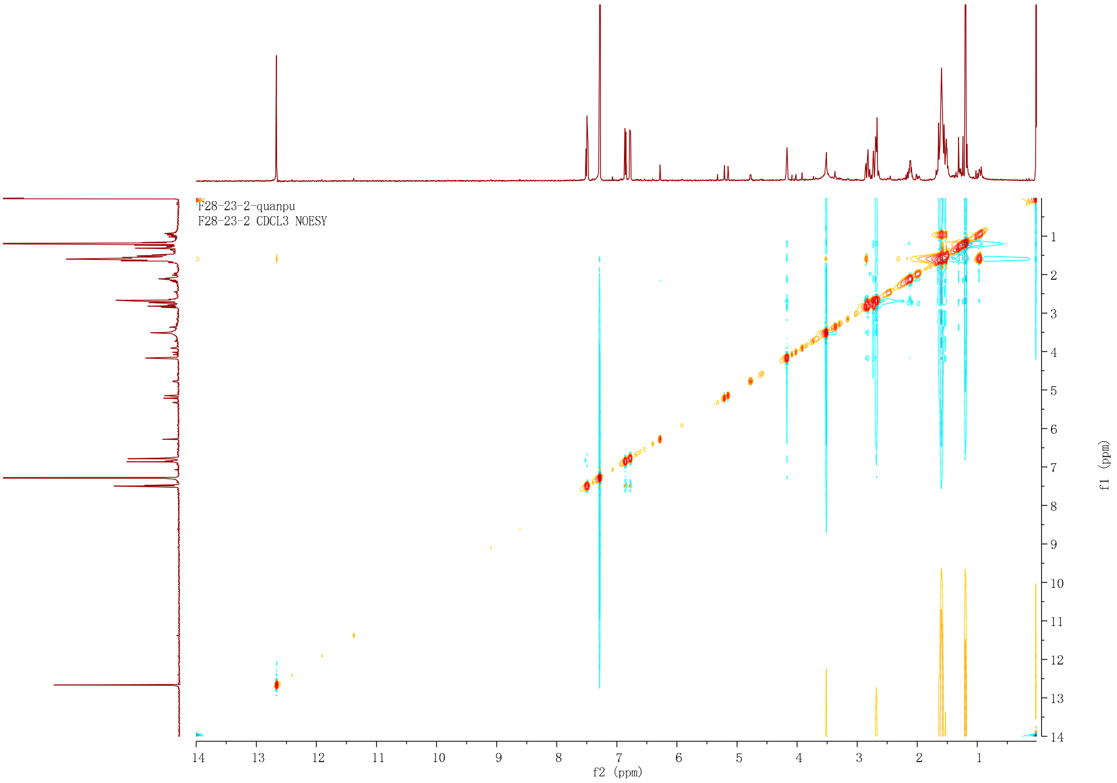

Figure S30. ^{1}H NMR (500 MHz, DMSO-d_6) spectrum of compound **6**

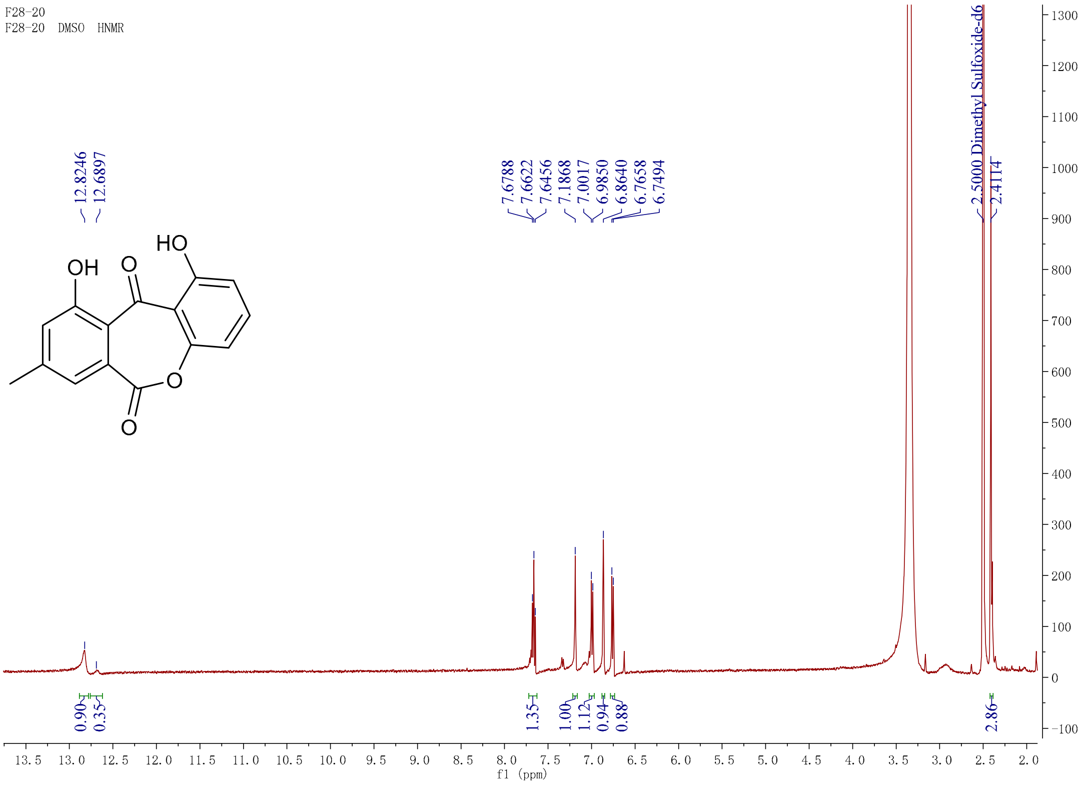

Figure S31. DEPTQ135 (125 MHz, DMSO-d_6) spectrum of compound **6**

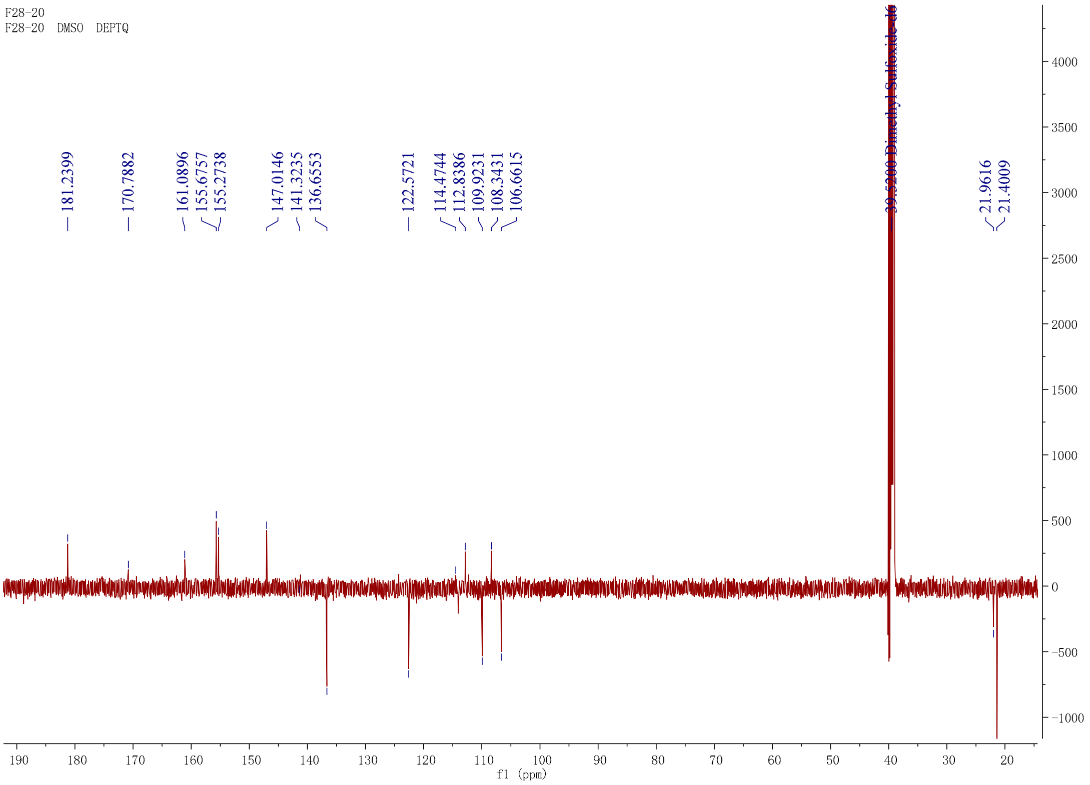

Figure S32. HSQC spectrum of compound **6**

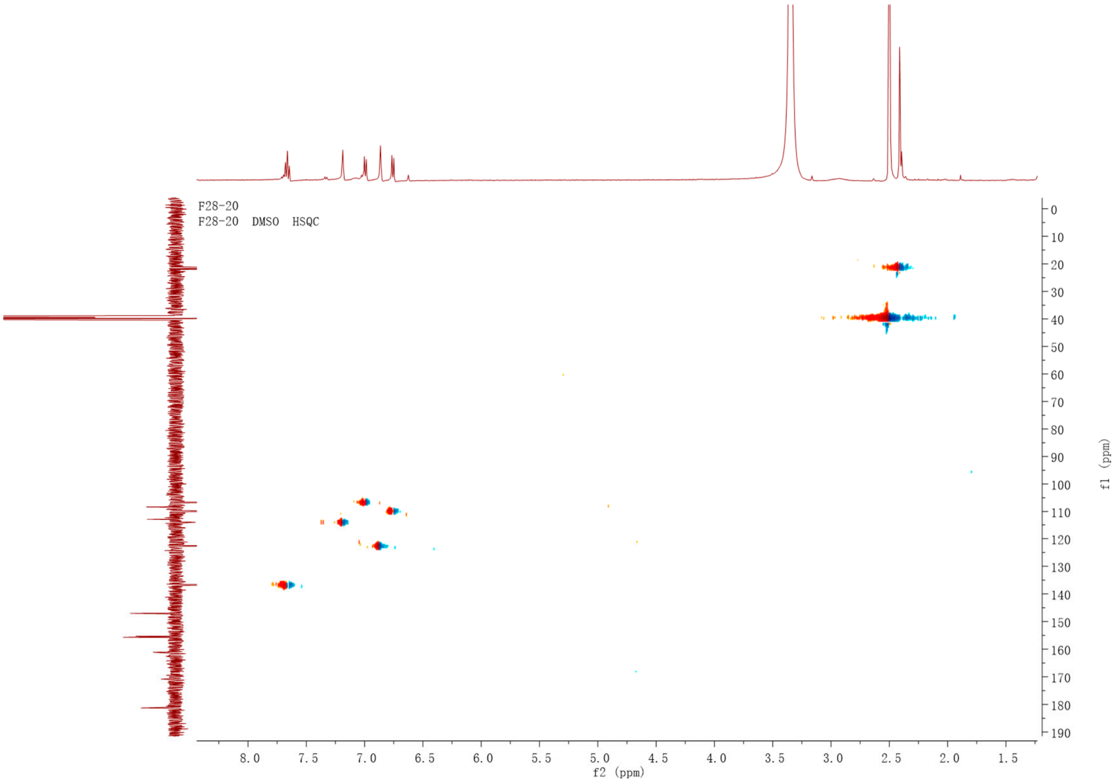

Figure S33. ^{1}H NMR (400 MHz, chloroform-*d*) spectrum of compound **7**

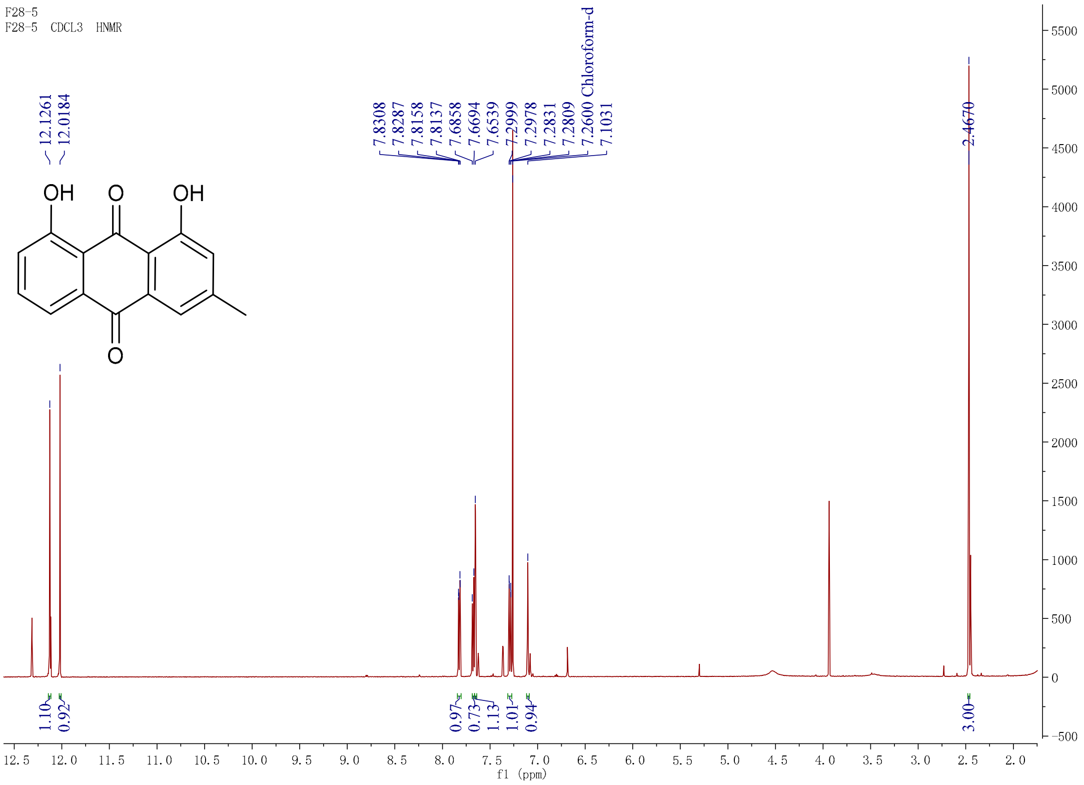

Figure S34. ^{1}H NMR (500 MHz, methanol-d_4) spectrum of compound **8**

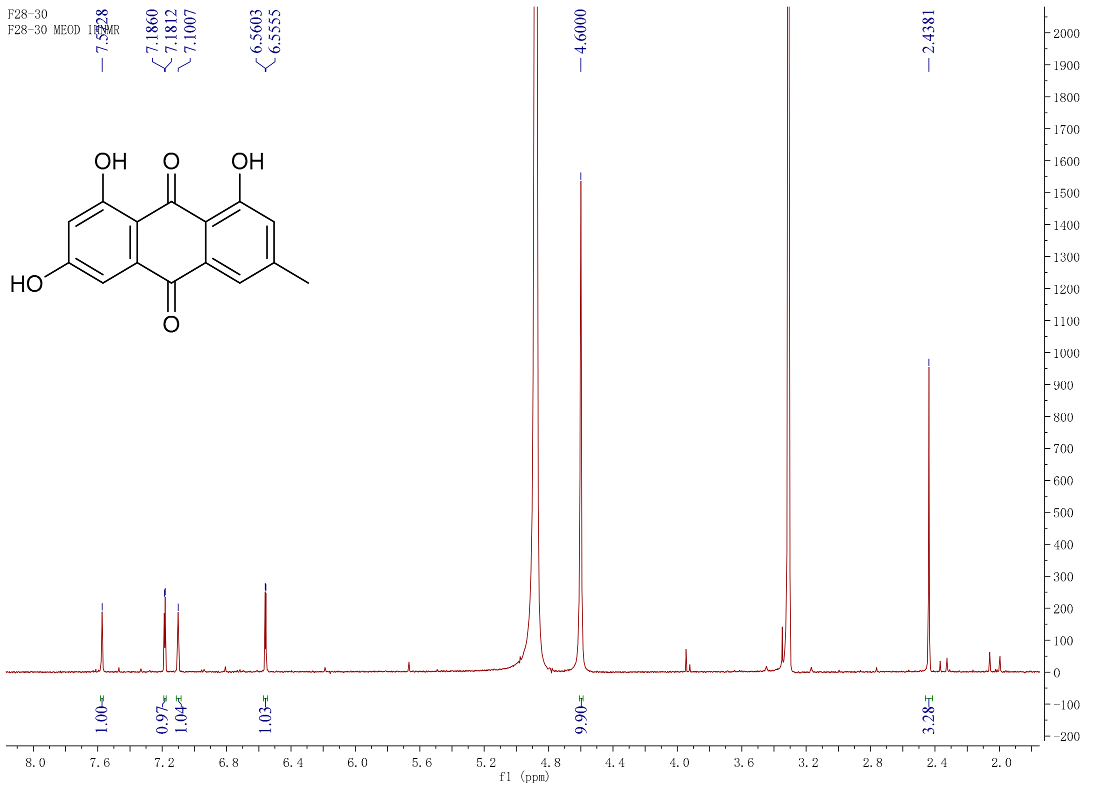

Figure S35. ^{13}C NMR (125 MHz, methanol-d_4) spectrum of compound **8**

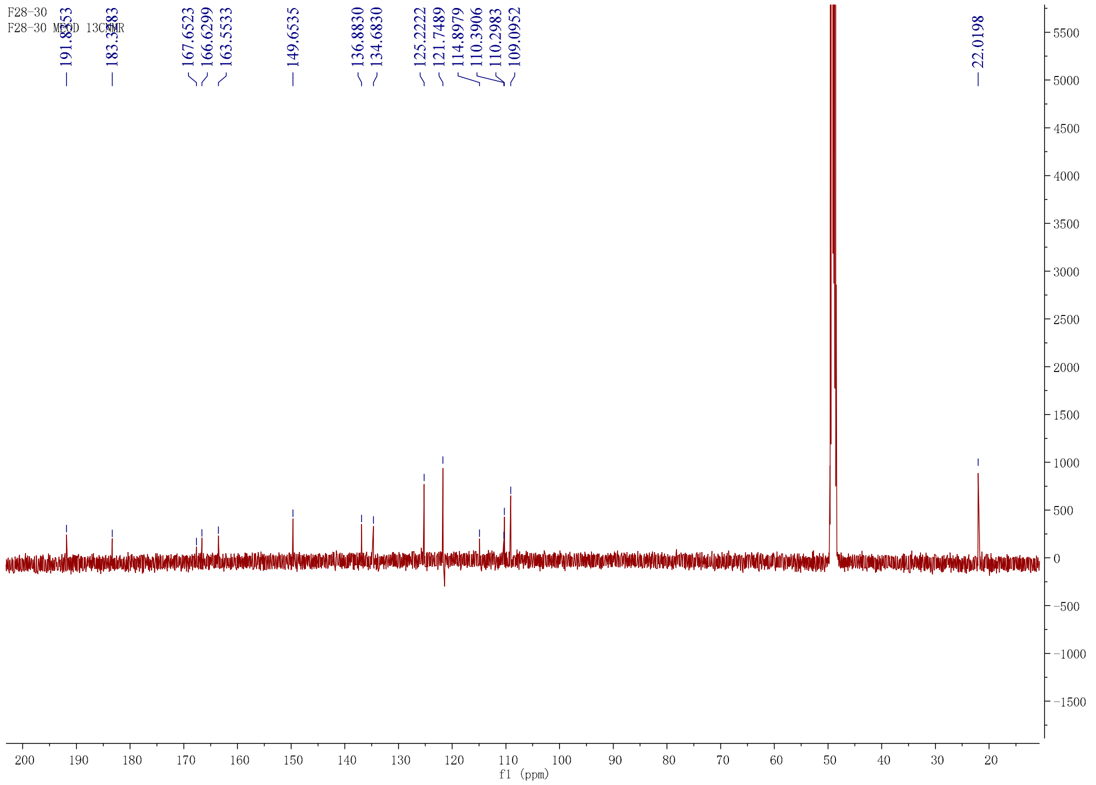

References

1. Pracht, P.; Bohle, F.; Grimme, S. Automated exploration of the low-energy chemical space with fast quantum chemical methods. *Physical chemistry chemical physics : PCCP* **2020**, *22*: 7169–7192.
2. Grimme, S. Exploration of chemical compound, conformer, and reaction space with meta-dynamics simulations based on tight-binding quantum chemical calculations. *Journal of chemical theory and computation* **2019**, *15*: 2847–2862.
3. Frisch, M.J.; Trucks, G.W.; Schlegel, H.B.; Scuseria, G.E.; Robb, M.A.; Cheeseman, J.R.; Scalmani, G.; Barone, V.; Petersson, G.A.; Nakatsuji, H.; Li, X.; Caricato, M.; Marenich, A.V.; Bloino, J.; Janesko, B. G.; Gomperts, R.; Mennucci, B.; Hratchian, H. P.; Ortiz, J. V.; Izmaylov, A.F.; Sonnenberg, J. L.; Williams; Ding, F.; Lipparini, F.; Egidi, F.; Goings, J.; Peng, B.; Petrone, A.; Henderson, T.; Ranasinghe, D.; Zakrzewski, V.G.; Gao, J.; Rega, N.; Zheng, G.; Liang, W.; Hada, M.; Ehara, M.; Toyota, K.; Fukuda, R.; Hasegawa, J.; Ishida, M.; Nakajima, T.; Honda, Y.; Kitao, O.; Nakai, H.; Vreven, T.; Throssell, K.; Montgomery Jr., J. A.; Peralta, J. E.; Ogliaro, F.; Bearpark, M. J.; Heyd, J. J.; Brothers, E.N.; Kudin, K.N.; Staroverov, V.N.; Keith, T.A.; Kobayashi, R.; Normand, J.; Raghavachari, K.; Rendell, A.P.; Burant, J.C.; Iyengar, S. S.; Tomasi, J.; Cossi, M.; Millam, J.M.; Klene, M.; Adamo, C.; Cammi, R.; Ochterski, J. W.; Martin, R.L.; Morokuma, K.; Farkas, O.; Foresman, J.B.; Fox, D.J. Gaussian 16 Rev. C.01: Wallingford, CT, **2016**.
4. Willoughby, P. H.; Jansma, M.J.; Hoye, T. R., A guide to small-molecule structure assignment through computation of ^{1}H and ^{13}C NMR chemical shifts. *Nature protocols* **2014**, *9*, 643–660.
5. Grimblat, N.; Zanardi, M.M.; Sarotti, A.M., Beyond DP4: An improved probability for the stereochemical assignment of isomeric compounds using quantum chemical calculations of NMR shifts. *The Journal of organic chemistry* **2015**, *80*, 12526–12534.
6. Pescitelli, G.; Bruhn, T., Good computational practice in the assignment of absolute configurations by TDDFT calculations of ECD spectra. *Chirality* **2016**, *28*: 466–474.
7. Lu, T.; Chen, F. Multiwfn: a multifunctional wavefunction analyzer. *J. Comput. Chem.* **2012**, *33*: 580–592.
